# Waiting Time Prioritisation for Public and Private Providers: Evidence From Hip Replacement Surgery

**DOI:** 10.1002/hec.70140

**Published:** 2026-08-08

**Authors:** Panos Kasteridis, Luigi Siciliani, Peter Sivey

**Affiliations:** ^1^ Centre for Health Economics University of York York UK; ^2^ Department of Economics and Related Studies University of York York UK

**Keywords:** capacity constraints, health care, prioritisation, provider behaviour, waiting times

## Abstract

Waiting times for planned health care treatments have been increasing in England and other OECD countries. One policy to reduce the impact of waiting times on patients' health is for doctors to prioritise patients according to their health status. This study tests the extent to which publicly‐funded patients in poorer health are prioritised on the list, and whether prioritisation differs between public and private providers. We use hospital administrative data in 2015–2021 on hip replacements in England. Our results provide evidence of inpatient waiting‐time prioritisation (from specialist addition to list to admission for surgery) based on pre‐operative health with a difference of at least 15 days between patients with lowest and highest pre‐operative health. The gradient becomes steeper as waiting times increase, and much steeper during COVID‐19. While outpatient waiting times (from referral to specialist visit) comprise a substantial proportion of total time waited (inpatient plus outpatient), we find limited evidence of outpatient waiting time prioritisation. Differences in inpatient waiting‐time prioritisation between public and private providers are mostly due to different casemix and shorter average waiting times by private providers. We also simulate the effect of prioritisation policies that reduce inpatient waiting times for patients with low pre‐operative health by 20% while simultaneously increasing it for those with high pre‐operative health. We find that these policies could generate health gains of 183 QALYs in a given year. We conclude that there is scope for improving prioritisation of patients on the waiting list.

## 1 Introduction

Long waiting times for hospital care are a major health policy issue in many countries (OECD 2020) that have been exacerbated by the COVID‐19 pandemic (Siciliani, Lafortune et al. 2025). Waiting times generate dissatisfaction amongst patients because they postpone health benefits from treatment and prolong suffering while waiting. Economists have argued that in the presence of an excess demand, waiting times act as a non‐monetary price that brings together the demand for and supply of health care (Lindsay and Feigenbaum 1984; Iversen 1993, 1997; Martin and Smith 1999, 2003; Martin et al. 2007). Some waiting times can help to reduce idle capacity (Iversen 1997) but this effect is likely to vanish quickly (Smet 2004; Siciliani, Stanciole, Jacobs et al. 2009). Long waiting times are an inefficient form of rationing as they impose a cost to patients that is not recovered by anyone else (Gravelle and Siciliani 2008a). Direct rationing is more efficient because it could reduce or eliminate long waiting times, but it is politically difficult to implement because it requires explicit refusal of health care to patients with low health benefits (Siciliani 2014).

Several policies aim at minimising the cost of waiting imposed on patients by prioritising patients on the list (OECD, 2013; 2020). Waiting time prioritisation arises in the health system at many levels. Emergency patients wait minutes or hours before being treated. For non‐emergency care, which is our focus, prioritisation can arise both across treatments and within treatments. Across treatments, patients tend to wait less for more urgent care, such as an elective coronary bypass, than for less urgent care, such as a hip or knee replacement. Waiting time prioritisation *across* and *within* treatments has been studied theoretically. Gravelle and Siciliani (2008a) show that a utilitarian regulator would set waiting times based on the patient's cost of waiting and the elasticity of demand to waiting times. In more detail, the optimal waiting time should be lower the higher the patient's marginal cost of waiting, but should be higher if the demand is more elastic to waiting times (because a higher elasticity implies that waiting times are more effective in reducing demand and bringing demand in line with the supply). The latter effect can reinforce or weaken the first effect depending on whether patients with higher marginal cost of waiting have lower or higher elasticity of demand.

Waiting time prioritisation also arises within treatments. Some countries like Canada and New Zealand explicitly have encouraged prioritisation through management tools that measure pre‐operative health or policy guidance (OECD 2013). Gravelle and Siciliani (2008b) provide a theoretical model of wait prioritisation for patients requiring the same treatment. They show that linear waiting time prioritisation based on observable health characteristics of the patients is welfare improving even in the presence of unobservable dimensions of health. They also show that wait prioritisation changes the composition of patients because fewer (more) patients with high (low) benefit are rationed. Direct rationing using observable characteristics, which they label “threshold rationing” would improve welfare even further, though such forms of rationing are rarely observed across health systems. In a companion paper, Gravelle and Siciliani (2009) show that optimal prioritisation across discrete patient groups receiving the same treatment depends again on patient cost of waiting and the demand elasticity to waiting time for each group.

Waiting time prioritisation across treatments is well documented because information on waiting times by treatment or procedures is routinely available across several countries (OECD 2020). In contrast, we know less about waiting time prioritisation within treatments. In this study, we investigate the extent to which doctors prioritise patients on the waiting list who require a hip replacement in publicly‐funded hospitals in England. The extent of prioritisation is theoretically indeterminate. As highlighted by the theoretical contributions above (e.g., Gravelle and Siciliani 2008a), there are at least two effects. On one hand, a higher cost of waiting for patients implies a reduction of waiting times for such patients. On the other hand, it is patients with higher marginal cost of waiting who may have a higher elasticity of demand, which would increase their waiting time and dampen the first effect. In our study, we test the extent to which patients with worse pre‐operative health (who are in more pain or less mobile), which closely relates to the patient cost of waiting, wait less than patients with better pre‐operative health.

To reduce waiting times, several countries like Denmark, England and Portugal have encouraged entry of private providers to treat publicly‐funded patients, often as part of policies that promote competition across providers (OECD 2013). In England, the proportion of publicly‐funded patients treated by private hospitals grew from 25% in 2015/16%–37% in 2020/21. Incentives between public and private providers differ. Economic theory suggests that private providers have stronger incentives to contain cost and treat less complex patients to maximise profits, while the effect on quality is in principle indeterminate (Brekke et al. 2012). Private providers tend to compete more aggressively on quality, including offering shorter waiting times to attract demand, but also have an incentive to skimp on quality to maximise profits. Private providers may also have different incentives to prioritise patients on the list. If private providers give a higher weight to profit relative to patients' health, this could dampen the incentive to prioritise patients on the list. We therefore test empirically whether provider behaviour in relation to waiting time prioritisation differs between public and private providers. Given that private providers differ in patients' casemix, we use (coarsened exact) matching techniques. We match patients using a range of characteristics, including pre‐operative health, gender, age, deprivation, diagnoses, and (the six most frequent) Elixhauser conditions.

We make several contributions to the literature that give insights into provider behaviour and how they prioritise patients on the list. First, we quantify the extent to which providers prioritise patients on the list for both the inpatient waiting time (from addition to list by the specialist to treatment) and the outpatient waiting time (from GP referral waiting time). While the inpatient waiting time is under direct control of hospitals, the outpatient waiting time is the result of the coordination between primary and secondary care providers. The earlier study by Gutacker et al. (2016) only focuses on inpatient waiting time. Using 2009‐14 data, it found 20 days difference between patients with high and low pre‐operative health, and no differences by socioeconomic status. Our approach captures more accurately the full patient experience, as the outpatient wait accounts for a substantial proportion of total wait from referral to treatment (more than one third in our data).^1^

Second, we test whether providers have a stronger incentive to prioritise patients on the list when waiting times are longer. If health benefits enter provider's utility function (in line with Ellis and McGuire 1986), prioritisation should become more pronounced when waiting times increase. Our empirical analysis exploits a period when waiting times gradually increased over time before the pandemic, and then suddenly increased during COVID‐19. In addition to pre‐operative health, which relates to the disutility from waiting, we also look at differences in waiting times by socioeconomic status. Several previous studies have shown that patients with higher socioeconomic status wait less for publicly‐funded care.^2^

Third, we are the first to compare waiting time prioritisation between public and private hospitals treating publicly‐funded patients. If private providers give a higher weight to profit relative to patients' health, this could dampen the incentive to prioritise patients on the list. We therefore contribute to the literature comparing waiting times and quality between public and private providers. Several studies have compared differences in quality between public and private providers across different countries (e.g., Shen 2002; Milcent 2005; Lien et al. 2008; Jensen et al. 2009; Moscelli, Gravelle, et al. 2018). Fewer studies have focused on differences in waiting times. Moscone et al. (2020) find no differences in waiting times between public and private providers in Italy, while Bjorvatn (2018) finds that they are shorter for private hospitals in Norway.

Fourth, we provide a framework to simulate the effect of different wait prioritisation policies and show that further redistributing waiting times from patients with high to low pre‐operative health can generate significant health gains. This framework is in line with literature linking pre‐operative health measures to the benefits of joint replacement (Price et al. 2020) and with theoretical predictions about waiting time prioritisation (Gravelle and Siciliani 2008b). The results are robust to different assumptions regarding the elasticity of demand with respect to waiting times, and the extent to which waiting times affect patient ability to benefit from health care.

## 2 Institutional Setting

The English National Health Service (NHS) provides universal access to health care, which is free at the point of use. Patients requiring hip replacement surgery first visit their general practitioner (GP) with symptoms of hip osteoarthritis and are subsequently referred to a hospital (McHugh et al. 2011; Dakin et al. 2020). There are two components of the waiting time, the outpatient waiting time for a specialist appointment with a consultant orthopaedic surgeon and the inpatient wait for subsequent surgery if deemed appropriate.

The NHS in England completed approximately 126,000 elective hip replacements per year prior to the pandemic (Krelle et al. 2021). Publicly‐funded patients can be treated by public hospitals (Nuffield Trust 2022) or private providers (known as the “independent sector”). About 25% of NHS patients in England were treated by private providers in 2015/16, which increased to 37% in 2020/21 (Peytrignet et al. 2022). Private providers can treat both publicly‐funded and privately‐funded patients, and some private providers treat only privately‐funded patients. About 25,000 hip replacements per year are privately‐funded and are not included in this study (PHIN 2022). Privately‐funded patients have to pay out of pocket if they do not have private health insurance. Only about 10% of patients hold private health insurance. Most private providers treating NHS patients are for‐profit, but some are not‐for‐profit.

The NHS has an 18‐week target (NHS England 2019) for any type of elective surgery, including hip replacement. Nearly 90% of patients were admitted within‐target in the early 2010s, falling to about 70% in 2019. During COVID‐19, elective care was severely disrupted. In March and April 2020, elective hip replacements fell to almost zero (Krelle et al. 2021). 58,000 fewer people had a hip replacement in 2020 compared to previous years. Only 59% of patients waited less than 18 weeks in 2022, and 56% for Trauma and Orthopaedics (NHS England 2022).

For some time, PROM‐based thresholds have been used locally in parts of the NHS at the referral stage, to determine if patients are suitable for surgery (Price et al. 2020). However, before COVID‐19, England did not have a national formal system for prioritising patients already on elective waiting lists for hip replacement. During the pandemic, the Federation of Specialty Surgical Associations produced guidelines (Federation of Specialty Surgical Associations 2021) suggesting that arthroplasty procedures (including joint replacement) should be prioritised if there was a risk of serious adverse consequences of delay and if an extended wait would prejudice the outcome of surgery. These guidelines could be interpreted as encouraging prioritisation on the basis of pre‐existing health status or capacity to benefit. However, it is unknown how these guidelines affected waiting time prioritisation in England in practice.

## 3 Data

Our main regression sample includes patients aged 18 or over who underwent a hip replacement surgery in an acute NHS hospital between April 2015 and March 2021, and for whom (a) we have information on their pre‐operative health and (b) we can measure both their inpatient and outpatient waiting times. Data are recorded based on financial years (between 1 April and 31 March). Our main regression sample for NHS hospitals is based on a number of criteria outlined in Supporting Information S1: Table A1 in Appendix A. We initially identify all hip replacement surgeries that took place during the study period using the procedure codes recorded in the Hospital Episode Statistics (HES) inpatient data. The identification algorithm includes all types of hip replacements but excludes bilateral operations and revisions.^3^ Over 70,000 hip replacements (excluding revisions) took place each year between 2015/16 and 2019/20 but the volume dropped dramatically to about 30,000 in 2020/21. We obtain information on pre‐operative health by linking hip replacement procedures identified in HES with PROMs data.

PROMs data have been collected nationally since 2009 for four elective surgical procedures, including hip replacement (NHS Digital 2022). Patients are invited to complete a pre‐operative and post‐operative PROMs questionnaire to measure their health status and health‐related quality of life.^4^ Each pre‐operative questionnaire includes a mixture of generic and condition‐specific questions. The Oxford hip score (OHS) is a condition‐specific instrument comprising 12 questions designed to assess a patient's experience of pain, ease of joint movement and ease of undertaking normal domestic activities such as walking or climbing stairs (Supporting Information S1: Appendix C). Each question scores on a five‐point scale, ranging from zero (severe problems) to four (no problems). Individual scores are added together to provide an overall score with zero indicating the worst possible score and 48 indicating the best possible score.

We link hip replacement procedures identified in HES with PROM records that have a completed pre‐operative OHS questionnaire and are linkable with HES.^5^ For 2015/16‐2019/20, a pre‐operative questionnaire was completed for 60%–67% of hip replacement surgeries. In 2020/21, the response rate fell to 38%. Inpatient waiting time is defined as the number of days between the specialist's decision to add a patient to the waiting list and the date the patient is admitted to hospital for surgery. We exclude inpatient waiting times longer than three standard deviations from each year's mean (1%).^6^ Of the records which can be linked with pre‐operative OHS, we exclude records with missing values on inpatient waiting times (mainly due to a missing or invalid date when the patient was added to the waiting list) and patient characteristics, totalling 11% of records over all 6 years, and a small number of records with age younger than 18.

We analyse separately hip replacements performed by independent sector (private) providers treating NHS‐funded patients (25% in 2015/16% and 37% in 2020/21). This is because waiting times for private providers are much shorter, which could have a significant impact on prioritisation.^7^

We use the outpatient HES database to identify outpatient appointments associated with the decision to add a patient to the inpatient waiting list. The process involves the following steps. The outpatient data provide information on the date of attendance of an appointment and the outpatient consultant's specialty, allowing us to identify relevant appointments as all outpatient appointments that (a) took place prior to the addition of the patient to the inpatient waiting list and (b) were led by a consultant whose specialty during the period of care was Orthopaedics.^8^ We organised the relevant outpatient appointments in spells as follows: an outpatient spell is defined as the set of a patient's outpatient appointments for which a referral (from a GP or other source) was received on the same date. A hip replacement record may be linked to one or more outpatient spells.^9^ Less than 0.5% of the outpatient appointments identified belong to spells with more than 10 appointments and are dropped from the sample as outliers. We consider the outpatient appointment that is nearest to the date of addition to the inpatient waiting list. If this appointment has taken place up to 3 days prior to the addition to the list, it is considered a match. Patients who do not have matched appointments are removed from the sample.

For the matched sample, we define the outpatient waiting time as the number of days between the referral date and the day the patient was added to the inpatient waiting list. We exclude outpatient waiting times that are longer than three standard deviations from each year's mean. We were able to match about 80% of hip replacements with an outpatient appointment (Supporting Information S1: Table A1).

The final regression sample consists of 21,192 hip replacement admissions in 2015/16, 24,059 in 2016/17, 22,166 in 2017/18, 23,233 in 2018/19, 18,956 in 2019/20 and 4414 in 2020/21. We split the OHS into nine groups coded as 0–4, 5–8, 9–12, 13–16, 17–20, 21–24, 25–28, 29–32, 33–48 to allow for a non‐linear relationship between pre‐operative health and waiting times which may emerge or change from year‐to‐year. The groups are computed as four‐points bands except for the top group (33–48), which is a residual group with highest pre‐operative health and a proportion of patients ranging between 2.9% and 3.8% (depending on the year, see Table 1), and the bottom group (0–4) with lowest level of pre‐operative health and a proportion of patients ranging between 4.1% and 5.6%. In a sensitivity analysis, we recode the 12 OHS questions as binary variables indicating difficulties in each domain, to assess the role of the individual domains comprising the OHS (Supporting Information S1: Appendix C).

**TABLE 1:** Summary statistics. Public providers (NHS Trusts).

*N*. of observations	2015/16	2016/17	2017/18	2018/19	2019/20	2020/21
	(21,192)	(24,059)	(22,166)	(23,233)	(18,956)	(4414)
Inpatient waiting time (mean, days)	90.120	103.846	109.362	120.487	120.798	219.466
Inpatient waiting time (median)	82	95	97	106	106	227
Outpatient waiting time (mean, days)	107.932	107.994	111.721	110.492	108.112	131.420
Outpatient waiting time (median)	48	50	51	48	49	70.5
Pre‐operative oxford hip score						
0–4	0.041	0.045	0.051	0.054	0.052	0.056
5–8	0.124	0.128	0.137	0.142	0.138	0.156
9–12	0.164	0.164	0.179	0.178	0.176	0.195
13–16 (reference group)	0.184	0.188	0.190	0.186	0.184	0.189
17–20	0.175	0.168	0.164	0.164	0.165	0.144
21–24	0.132	0.132	0.129	0.120	0.127	0.112
25–28	0.092	0.090	0.077	0.083	0.079	0.076
29–32	0.051	0.051	0.045	0.043	0.046	0.039
33–48	0.038	0.035	0.029	0.031	0.033	0.033
Symptoms duration						
Less than a year	0.127	0.122	0.107	0.104	0.099	0.059
1–5 years (ref. group)	0.690	0.692	0.697	0.704	0.708	0.751
6–10 years	0.115	0.117	0.124	0.121	0.125	0.116
> 10 years	0.069	0.069	0.072	0.070	0.069	0.075
Male	0.404	0.402	0.402	0.407	0.400	0.390
Age						
18–44	0.031	0.030	0.030	0.029	0.028	0.039
45–54	0.085	0.087	0.090	0.083	0.082	0.093
55–64 (ref. group)	0.205	0.203	0.200	0.205	0.215	0.220
65–74	0.353	0.357	0.360	0.353	0.346	0.355
75–84	0.277	0.270	0.267	0.277	0.276	0.249
≥ 85	0.048	0.053	0.052	0.053	0.052	0.045
Ethnicity						
White (ref. group)	0.892	0.890	0.882	0.881	0.878	0.849
Mixed	0.002	0.002	0.002	0.002	0.002	0.001
Asian	0.005	0.005	0.006	0.005	0.005	0.009
Black	0.007	0.006	0.007	0.007	0.007	0.007
Other ethnicity	0.004	0.005	0.005	0.004	0.005	0.003
Missing ethnicity	0.090	0.092	0.099	0.101	0.104	0.130
Rural (ref group: Urban)	0.253	0.270	0.274	0.278	0.274	0.294
Emergency admissions^a^						
0 (ref. group)	0.919	0.913	0.910	0.907	0.903	0.922
1	0.064	0.068	0.072	0.075	0.076	0.059
≥ 2	0.017	0.019	0.018	0.018	0.022	0.018
Primary diagnosis^b^						
Coxarthr., unsp. (ref. group)	0.740	0.755	0.768	0.766	0.773	0.750
Other primary coxarthrosis	0.107	0.095	0.079	0.084	0.084	0.101
Polyarthrosis, unspecified	0.061	0.067	0.073	0.073	0.070	0.061
Osteonecrosis, unspecified	0.010	0.011	0.011	0.011	0.009	0.012
Primary coxarthrosis, bilateral	0.017	0.010	0.007	0.007	0.007	0.007
Secondary diagnosis^c^						
Hypertension, uncomplicated	0.482	0.481	0.493	0.492	0.489	0.440
Obesity	0.154	0.173	0.200	0.227	0.256	0.249
Chronic pulmonary disease	0.162	0.167	0.172	0.178	0.179	0.169
Diabetes, uncomplicated	0.109	0.110	0.111	0.112	0.111	0.100
Cardiac arrhythmias	0.092	0.097	0.107	0.109	0.109	0.094
Hypothyroidism	0.080	0.088	0.089	0.094	0.101	0.089
Depression	0.060	0.066	0.077	0.081	0.086	0.096
Renal failure	0.068	0.072	0.077	0.079	0.085	0.071
Rheumatoid arthritis	0.055	0.061	0.065	0.065	0.066	0.073
Valvular disease	0.028	0.032	0.036	0.038	0.042	0.029
Index of multiple deprivation (IMD) quintile						
1 (ref. group—least deprived)	0.221	0.225	0.227	0.228	0.229	0.250
2	0.227	0.233	0.232	0.231	0.228	0.245
3	0.211	0.214	0.216	0.218	0.213	0.227
4	0.185	0.180	0.182	0.176	0.184	0.167
5 (most deprived)	0.155	0.148	0.144	0.147	0.146	0.111
Referral source						
GP referral (ref. group)	0.773	0.774	0.755	0.749	0.740	0.732
Responsible consultant	0.033	0.029	0.028	0.027	0.025	0.026
Other	0.194	0.197	0.217	0.224	0.235	0.243
Number of outpatient appointments						
1 (ref. group)	0.661	0.657	0.653	0.639	0.644	0.580
2	0.153	0.154	0.155	0.165	0.160	0.189
3	0.076	0.077	0.075	0.080	0.077	0.089
> 3	0.110	0.113	0.117	0.117	0.119	0.142

*Note:* Mean values for all listed variables (except for the two labelled “median”) over six financial years.

^a^ Emergency admissions in the year preceding a hip replacement surgery.

^b^ Indicators for the most frequent primary diagnosis at admission and an indicator for any other primary diagnosis. We only show the most common five primary diagnoses; other diagnoses included in the models are: other secondary coxarthrosis, other dysplastic coxarthrosis, gonarthrosis (unspecified), arthritis (unspecified), other post‐traumatic coxarth., other.

^c^ Indicators for each of the Elixhauser morbidities recorded in the year preceding the hip replacement surgery. We only show the most common 10 secondary diagnoses; other diagnoses included: fluid & electrolyte disorder, alcohol abuse, other neurological disorder, congestive heart failure, peripheral vascular disorder, deficiency anemia, solid tumor w/o met., liver disease, coagulopathy, diabetes complicated, peptic ulcer, weight loss, pulmonary circulation disorder, metastatic cancer, lymphoma, drug abuse, paralysis, psychoses, hypertension (complicated), blood loss anaemia.

We control for various patient characteristics including patients' sex, age, ethnicity, rurality, deprivation, and year and month of admission. We construct six age groups: 18–44 years old, 45–54, 55–64, 65–74, 75–84 and 85 years or above. Rurality is captured by a binary variable indicating whether a patients' output area (average population of 300 residents) falls within towns, villages, hamlets and isolated dwellings. To proxy socioeconomic status, we use the 2019 Index of Multiple Deprivation (IMD) quintile for the patient's area of residence (Lower Super Output Area with average population of 1500 inhabitants) as an indicator of area‐level deprivation. We account for prior health care utilisation by including a categorical variable indicating whether a patient had zero, one, or more than one emergency admissions in the year prior to their joint replacement surgery. To account for the complexity of patients' condition, we include dummy variables indicating the type of primary diagnosis at admission, plus each of the conditions of the Elixhauser index.^10^ We use the consultant code to account for the specialist doctor who had primary responsibility for the patient during their stay (usually the surgeon). Finally, for the analysis of outpatient waiting times we construct two additional variables: dummy variables indicating the source of referral (GP, responsible outpatient consultant, other) and the number of appointments in the outpatient spell (1, 2, 3, 4 or more).

Supporting Information S1: Table A2 compares the original sample, non‐respondents to the pre‐operative health questionnaire, and the final estimation sample for NHS hospitals. The table shows that the final sample is broadly representative (within 1‐2 percentage points) of both the original sample and the sample of non‐respondents in terms of age, sex, rurality, primary and secondary diagnoses, and IMD deprivation measures.

Supporting Information S1: Table A3 extends the comparison between non‐respondents and the final sample to all years and suggests limited selection on observed characteristics over the study period. However, some larger differences are observed in inpatient waiting times, with non‐respondents having longer waits on average (e.g., mean 96 days in 2015/16 compared to 90 days in the final sample; mean 227 days in 2020/21 compared to 219 days in the final sample). We address this issue more formally in a robustness check section using an inverse probability weighting approach.

Selection would bias the estimated prioritisation gradient only if PROMs completion is jointly related to both waiting time and unobserved pre‐operative health. As OHS is not observed for non‐respondents, we cannot assess whether such joint selection is present, nor determine the direction or magnitude of any resulting bias. However, the similarity in observables provides reassurance that respondents and non‐respondents are broadly comparable.

When comparing respondents and non‐respondents, patient characteristics also look reasonably similar. In 2019/20, the proportion of male patients is 40.0% in the final sample and 40.7% in the non‐respondents sample, the proportion of patients in the 65–74 age group is 34.6% and 33.2%, the proportion of white patients is 87.8% and 84.4%, the proportion of rural patients is 27.4% and 26.7%; and the proportion of patients with no emergency admissions is 90.3% and 88.1%. The results are also comparable for 2020/21 despite the higher rate of missing observations on pre‐operative health (62% relative to 40% in the previous year).

## 4 Methods

Our regression model of waiting times is:

(1) $$W_{ijt}={\left(H_{ijt}⊗d_{t}\right)}^{\prime }\;\beta +{\left(X_{ijt}⊗d_{t}\right)}^{\prime }\gamma +{\left(s_{j}⊗d_{t}\right)}^{\prime }\delta +\varepsilon_{ijt},$$

where $W_{ijt}$ is the inpatient or outpatient waiting time of patient *i* treated by specialist *j* at time *t*; $H_{ijt}$ is a vector of indicators of pre‐operative health as measured by the Oxford Hip Score; $X_{ijt}$ is a vector of patients characteristics (e.g., age, sex, ethnicity, primary and secondary diagnosis, year and month of admission, etc.); $s_{j}$ are specialist fixed effects that control for differences in waiting times across specialists (surgeons) within hospitals which arise from unobserved supply factors. $d_{t}$ is a vector of year fixed effects. Finally, $u_{ijt}$ is an idiosyncratic error term. To capture how prioritisation may change over time, the model is estimated by fully interacting all the variables with year dummies $d_{t}$. This specification allows the effects of pre‐operative health and patient characteristics to vary flexibly across years and controls for specialist‐by‐year differences in supply and demand factors. The Kronecker product ⊗ creates all interactions between the patient characteristics and specialist dummies, and the year dummies.

The key coefficients of interest are β that capture the degree of waiting time prioritisation by pre‐operative health status in each year, conditional on patient and provider characteristics. γ and δ are the equivalent coefficients for other patient characteristics and specialist fixed effects. We estimate the model in (1) by Ordinary Least Squares with standard errors clustered at the specialist level.^11^

Including patient characteristics is important to control for factors that may also influence waiting times and be correlated with the Oxford Hip Score, which captures pre‐operative health related to pain and lack of mobility due to problems with the hip. Variation in waiting time according to other patient characteristics can be interpreted as other forms of prioritisation (e.g., age), inequalities (such as deprivation), or clinical factors that can slow down patient's journey (e.g., co‐morbidities which preclude treatment in certain types of facilities). The inclusion of specialist fixed effects implies that our estimates can be interpreted as the degree of prioritisation for a given specialist, and we therefore control for any correlation between pre‐operative health and waiting times at the specialist level (for instance, if patients in worst health are treated by certain specialists in hospitals with low waiting times).

To assess whether patients treated by private providers are prioritised differently from patients treated by public providers, we match NHS and private providers and estimate regressions separately on the matched samples. We perform coarsened exact matching (CEM), which involves the following steps: (a) each matching variable is coarsened into substantively meaningful groups; (b) coarsened covariate patterns (strata) are created representing unique combinations of values across all coarsened covariates; (c) each treated and control unit is assigned into one of the strata based on their coarsened values (exact matching on the coarsened variables); (d) any stratum that does not contain at least one treated and one control unit is pruned; (e) the original (uncoarsened) variables of the matched samples are used in separate regression analyses to determine the effects on waiting times.

We perform two types of matching. First, we match at the patient level on selected patient characteristics (OHS group, gender, age, deprivation, the three most frequent primary diagnosis, and the six most frequent Elixhauser conditions). This allows us to compare similar patients treated in public and private hospitals and to adjust for differences in casemix. We use k:m matching, which allows for a different number of treated and control units within each stratum. Matching weights are used in the regressions to adjust for strata size. Second, we conduct provider‐level matching using a k:k approach based on average inpatient waiting times, to compare NHS and private hospitals operating under similar capacity constraints. Regressions in both cases are estimated at the patient level on the matched samples.

Both matching approaches rely on the assumption of conditional independence: once we match on observed patient or provider characteristics, remaining differences in waiting time prioritisation can be attributed to provider type, conditional on no unobserved confounding. For patient‐level matching, this assumes that selection into public or private treatment is captured by the matched variables. For provider‐level matching, we assume that matching on average waiting times accounts for relevant provider‐level differences. While we cannot fully rule out unobserved confounding, the richness of the covariates and the use of two complementary strategies strengthen the credibility of our comparisons and reduce the risk that findings are driven by differences in casemix or capacity constraints.

For both matching approaches, we assess the balance achieved by the matching using unidimensional measures of imbalance such as differences in means and percentiles for each variable as well as the multivariate L1 distance statistic (Iacus et al. 2009) that measures the balance of the joint distribution of variables (global balance).

## 5 Results

### 5.1 Descriptive Statistics

Table 1 provides summary statistics for public providers. The mean inpatient waiting times increased from 90 days in 2015/16–121 days in 2019/20, and then sharply to 219 days in 2020/21 after the onset of COVID‐19. Outpatient waiting times varied little between 2015/16 and 2019/20 with a mean of 108–112 days but increased to 131 days in 2020/21. The median outpatient waiting time is about half of the mean outpatient waiting time. Most patients experience relatively low outpatient waiting time while a small proportion of patients experience a very long wait.^12^ One possible explanation is that some patients may have complications or experience other health problems that require postponing the surgery. Others may face challenges in their personal life that may make it difficult to proceed with surgery. Some of the less complex patients may also experience long delays to ensure that surgery is appropriate for them. This finding that the mean waiting times is above the median is not specific to hip replacement but it is very common across many procedures within the English NHS and across health systems (OECD, 2013; 2020).

The patient composition was generally stable across years, even in 2020/21 when the sample size is much smaller. About 52% of patients have a preoperative OHS between 9 and 20 points, 40% are male, and 35% are between 65 and 74 years old. 85% have white ethnicity in 2020/21, about 2‐4 percentage points lower than the pre‐pandemic period. Less than 30% of patients reside in rural areas. At least 90% of patients have no emergency admissions in the year prior to surgery, and about 2% have more than one.

Different types of arthritis of the hip account for more than 90% of admissions (unspecified coxarthrosis 74%–77%; other primary coxarthrosis 8%–10% and polyarthrosis for 6%–7%). Less than 16% of patients are in the most deprived quintile in 2015/16 and this percentage drops to 11% in 2020/21, when 50% of patients are in the first two (least deprived) quintiles. The referral source from a GP was at least 73% of the outpatient appointments. 34% of patients in 2015/16 had two or more appointments.

### 5.2 Regression Results. Inpatient Waiting Times

To assess how waiting time prioritisation changes over time, we estimated a linear model^13^ in which all variables are interacted with year dummies. Supporting Information S1: Table A4 in the Appendix A provides the full set of regression estimates. The first column reports the estimates for the baseline year, while subsequent columns present the interaction terms for each variable with the year dummies. These interactions indicate the change of each variable relative to the baseline in each subsequent year.

Our key findings are presented in Table 2, which focuses on the OHS categories and reports their marginal effects for each year. These marginal effects are calculated by summing the baseline estimate (first column of Supporting Information S1: Table A4) with the corresponding interaction term for the specific year (relevant column of Supporting Information S1: Table A4). Figure 1 shows the inpatient waiting times across different OHS categories, conditional on other variables. The plotted points represent the average waiting time for each OHS category with vertical bars indicating the 95% confidence intervals.

**TABLE 2:** Marginal effects for oxford hip score: inpatient waiting times.

*N*. of observations	2015/16	2016/17	2017/18	2018/19	2019/20	2020/21
	(21,192)	(24,059)	(22,166)	(23,233)	(18,956)	(4414)
Pre‐operative oxford hip score						
0–4	−6.50***	−8.25***	−6.57***	−11.42***	−11.29***	−18.38***
	(2.00)	(1.80)	(1.79)	(1.72)	(1.94)	(4.18)
5–8	−2.72**	−2.98**	−4.02***	−4.92***	−8.64***	−22.95***
	(1.34)	(1.24)	(1.26)	(1.22)	(1.38)	(2.90)
9–12	−2.31*	−1.83	−3.13***	−2.54**	−2.92**	−4.37
	(1.23)	(1.14)	(1.16)	(1.14)	(1.28)	(2.70)
13–16 (reference group)						
17–20	1.35	1.83	2.06*	2.00*	1.85	5.23*
	(1.21)	(1.14)	(1.19)	(1.17)	(1.30)	(2.95)
21–24	2.97**	3.49***	3.32***	2.64**	3.09**	3.26
	(1.31)	(1.22)	(1.28)	(1.28)	(1.40)	(3.16)
25–28	5.42***	3.53	4.81***	4.94***	4.36***	11.88***
	(1.47)	(1.38)	(1.51)	(1.45)	(1.63)	(3.66)
29–32	4.66**	4.90***	6.90***	7.69***	6.05***	29.38***
	(1.82)	(1.70)	(1.85)	(1.84)	(2.00)	(4.62)
33–48	8.07***	10.64***	12.13***	9.45***	8.26***	10.07**
	(2.06)	(1.99)	(2.25)	(2.13)	(2.31)	(5.08)

*Note:* Standard errors in parentheses. Marginal effects are presented for the Oxford Hip Score categories only. The model from which these marginal effects are derived includes patient characteristics listed in Table 1, consultant fixed effects, and year and month dummy variables. Patient characteristics and consultant fixed effects are interacted with year dummy variables. Full estimates for all covariates are provided in Appendix, Supporting Information S1: Table A4.

^*^ *p* < 0.1.

^**^ *p* < 0.05.

^***^ *p* < 0.01.

**FIGURE 1:**
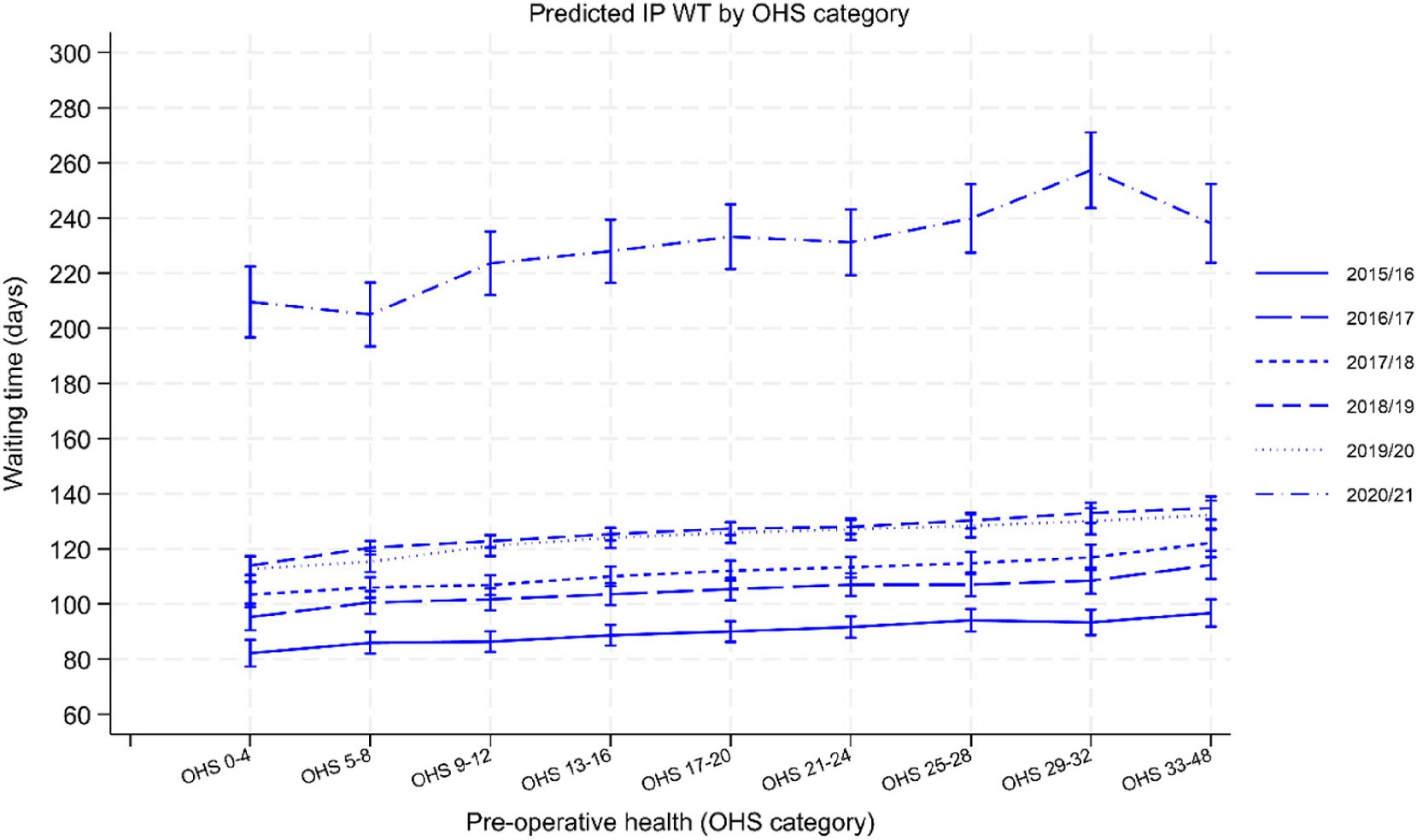
Predicted inpatient waiting times by Oxford Hip Score category. The plotted points represent the average predicted waiting time for each OHS category. The vertical bars indicate the 95% confidence intervals.

Table 2 and Figure 1 provide evidence of waiting time prioritisation. Patients with better pre‐operative health (larger values of OHS) wait longer. In 2015/16, relative to the reference group (13–16 OHS points), patients achieving 29–32 (33–48) points on the OHS scale wait about 5 (8) days longer and patients with 5–8 (0–4) points wait about 3 (7) days shorter. This amounts to at most 15 days difference between the lowest and highest pre‐operative health.

The gradient becomes generally steeper every year in the pre‐pandemic period. Patients with worst pre‐operative health wait less relative to the reference group by 8 days in 2016/17, 7 days in 2017/18, and 11 days in 2018/19 and 2019/20. In 2020/21, waiting time prioritisation is even steeper. Patients with the worst pre‐operative health wait 18 days less, while those with 29–32 points wait 29 days longer compared to the reference group. The difference in waiting time between the lowest and highest pre‐operative health similarly increases from 15 days in 2015/16–19 days in 2017/18, and 21 days in 2018/19, and 28 days in 2020/21.

However, this gradual, consistent change over time, is not dramatic enough to be detected by most pairwise tests for the change in each coefficient from year to year. The Wald tests presented in Supporting Information S1: Table A5 in Appendix A reveal a statistically significant difference when comparing the coefficients of the 5–8 and 29–32 OHS categories for 2019 with those for 2020. For most of the other pairwise comparisons of years, the differences in coefficients are statistically insignificant.

There is generally no income deprivation gradient in the pre‐pandemic period (Supporting Information S1: Table A4). The deprivation gradient becomes remarkably steeper in 2020/21 with those in the most deprived quintile waiting more than 20 days longer compared to those in the least deprived quintile. We generally find no differences in waiting times by ethnicity.

Several other patient characteristics are also associated with waiting time (Supporting Information S1: Table A4). For given pre‐operative health, younger patients tend to wait longer.^14^ Patients admitted to hospital as an emergency within the last year wait significantly less than those who had not.^15^ Longer symptom duration is associated with longer waiting time.^16^ Relative to unspecified coxarthrosis, patients with osteonecrosis have a shorter waiting time.

### 5.3 Outpatient Waiting Times

Table 3 provides the estimates for the outpatient waiting times. This specification also controls for the source of outpatient referral (GP vs. responsible consultant vs. other) and number of outpatient appointments. The results provide some evidence of prioritisation in outpatient waiting time with respect to pre‐operative health, though this is less pronounced relative to inpatient waiting time and it is concentrated amongst patients with better pre‐operative health. In 2016/17–2018/19, patients with better pre‐operative health (33–48 OHS points) wait between 10 and 12 days longer than the reference group. This effect is less pronounced in 2015/16 and 2019/20, and absent in 2020/21. In contrast, there is only weak evidence that patients with low pre‐operative health differ in outpatient waiting time relative to the reference group. The Wald tests presented in Supporting Information S1: Table A6 do not detect changes in the coefficients from year to year.

**TABLE 3:** Marginal effects oxford hip score: outpatient waiting times.

*N*. of observations	2015/16	2016/17	2017/18	2018/19	2019/20	2020/21
	(21,192)	(24,059)	(22,166)	(23,233)	(18,956)	(4414)
Pre‐operative oxford hip score						
0–4	−3.38	1.29	−6.03**	−5.12*	−0.89	−8.87
	(3.39)	(3.05)	(3.02)	(2.91)	(3.28)	(7.07)
5–8	−6.34***	−2.49	−3.33	−2.72	−0.63	−9.01*
	(2.27)	(2.09)	(2.13)	(2.07)	(2.33)	(4.90)
9–12	−2.78	−1.74	2.38	−2.87	−2.28	−2.62
	(2.08)	(1.93)	(1.97)	(1.94)	(2.16)	(4.58)
13–16 (reference group)						
17–20	2.60	3.18*	5.03**	2.20	3.48	−1.56
	(2.04)	(1.92)	(2.01)	(1.97)	(2.20)	(5.00)
21–24	3.76*	8.01***	5.89***	1.42	3.91*	1.22
	(2.21)	(2.06)	(2.16)	(2.16)	(2.37)	(5.34)
25–28	2.13	7.45***	5.78**	1.93	3.67	3.05
	(2.49)	(2.34)	(2.56)	(2.45)	(2.76)	(6.20)
29–32	3.17	7.06	7.32**	9.63***	8.56**	−9.29
	(3.09)	(2.88)	(3.14)	(3.12)	(3.39)	(7.81)
33–48	7.22**	10.92***	11.58***	9.62***	7.96**	−2.16
	(3.48)	(3.37)	(3.81)	(3.60)	(3.91)	(8.59)

*Note:* Standard errors in parentheses; Marginal effects are presented for the Oxford Hip Score categories only. The model from which these marginal effects are derived includes patient characteristics listed in Table 1, consultant fixed effects, and year and month dummy variables. Patient characteristics and consultant fixed effects are interacted with year dummy variables. Full estimates for all covariates are provided in the Appendix, Supporting Information S1: Table A7.

^*^ *p* < 0.1.

^**^ *p* < 0.05.

^***^ *p* < 0.01.

Supporting Information S1: Table A7 provides the full regression results. In relation to income deprivation and ethnicity, we generally find no differences in outpatient waiting time neither before or after COVID‐19. Along the patient pathway, we find that patients who require multiple outpatient appointments have much longer outpatient waiting time than patients with only a single GP appointment. For example, in 2015/16 patients with two (three) outpatient appointments waited 87 (191) days longer relative to those with one appointment, and those with more than four waited almost a year longer.

Some other patient characteristics are also associated with outpatient waiting time. Younger patients generally wait longer. Patients who have had more than two emergency admissions experience shorter waits. Patients with symptom duration less than a year wait less than those whose symptoms last 1–5 years by about 15–18 days depending on the year. Similar to the inpatient results, patients with osteonecrosis as a primary diagnosis wait less than patients with coxarthrosis. There is no consistent pattern on the effects of co‐morbidities (secondary diagnoses) on outpatient waiting time. Relative to patients referred by the GP, patients referred by a consultant wait 20 days longer.

Given that patients with better pre‐operative health (higher OHS points) are more likely to have multiple appointments and more appointments are associated with longer outpatient waiting times, patients with better pre‐operative health tend to have a longer outpatient waiting time (everything else given). This is shown in Supporting Information S1: Table A8 (“partial adjustment” model): outpatient waiting time prioritisation is more pronounced when we omit the number of appointments and source of referral. There is a difference of 20–34 days between patients with the highest OHS and the reference group across all years except 2020–21.

Our sample is heterogeneous in terms of number of outpatient appointments and source of referral. Many patients have a straightforward pathway. 77% of patients are referred by a GP and 66% have only one outpatient appointment. Supporting Information S1: Table A8 (“restricted sample” model) shows that GPs do not prioritise patients based on pre‐operative health for patients with a single outpatient appointment (about 50% of the sample). The coefficients are quantitatively small and only occasionally statistically significant at 5% level. This further corroborates the finding that there is very limited wait prioritisation for an outpatient appointment.

### 5.4 Private Providers

In this section, we test whether patients treated by private providers are prioritised differently. Private providers treated 25% of patients in 2015/16 increasing to 37% in 2020/21. Waiting times are generally shorter for private providers, for both the inpatient and outpatient component. In 2018/19 (2019/20), inpatient waiting time was on average 59.1 (66.7) days for private providers and 120.5 (120.8) days for public providers (Supporting Information S1: Appendix D). In 2020/21 the wait increased to 129.4 days for private providers and 219.5 days for public providers.

Given that patient casemix can differ between public and private providers, differences in wait prioritisation could reflect differences in casemix. We use matching approaches to compare inpatient wait prioritisation across matched samples. Table 4 presents the regression results on samples created by carrying out a “k‐to‐m” coarsened exact matching on patient characteristics. For private providers, it suggests that patients with better pre‐operative health wait generally longer. For example, patients with an OHS of 25–28 wait 5–9 days longer than the reference group between 2015/16 and 2019/20 (except for 2017/18). Patients with an OHS of 33–48 wait at least 6 days longer each year, and this increases to 31 days in 2020‐21. In 2017/18 and 2018/19, the difference in inpatient wait between patients with highest and lowest pre‐operative health was 19 days, which increased to 24 days in 2019/20. The difference increases further to 40 days in 2020/21 between patients with highest and lowest pre‐operative health.

**TABLE 4:** Inpatient waiting time. Matched sample (k:m).

a. Private providers (independent sector)						
	2015/16	2016/17	2017/18	2018/19	2019/20	2020/21
*N*. of observations	(6262)	(7446)	(7020)	(9555)	(8453)	(1651)
Mean wait time (days)	52.1	50.7	51.2	58.4	65.7	126.6
Pre‐operative oxford hip score						
0–4	0.93	2.57	12.15**	−8.47*	−11.54**	−9.57
	(6.56)	(5.77)	(5.79)	(4.81)	(5.48)	(12.69)
5–8	0.44	2.08	−3.11	−1.76	−4.88*	−15.57**
	(3.05)	(2.78)	(2.82)	(2.35)	(2.58)	(6.05)
9–12	1.27	2.25	−1.15	−0.57	−3.81*	−1.48
	(2.55)	(2.31)	(2.30)	(1.97)	(2.11)	(5.00)
13–16 (reference group)						
17–20	−0.91	2.77	2.48	0.16	1.17	15.28***
	(2.34)	(2.13)	(2.14)	(1.84)	(1.97)	(5.07)
21–24	1.69	4.34*	4.41*	4.22**	6.89***	7.30
	(2.46)	(2.24)	(2.30)	(1.91)	(2.10)	(5.42)
25–28	6.43**	5.74**	2.87	8.58***	5.34**	9.61
	(2.66)	(2.50)	(2.58)	(2.19)	(2.30)	(6.36)
29–32	4.84	4.83	3.44	5.89**	4.86*	−8.13
	(3.18)	(3.00)	(3.26)	(2.70)	(2.83)	(8.30)
33–48	5.89*	11.61***	7.21*	10.66***	12.81***	30.95***
	(3.58)	(3.40)	(3.83)	(3.25)	(3.30)	(9.13)

**b. Public providers (NHS trusts)**						
	**2015/16**	**2016/17**	**2017/18**	**2018/19**	**2019/20**	**2020/21**
***N*. of observations**	**(12995)**	**(14832)**	**(13654)**	**(15086)**	**(12057)**	**(2014)**
**Mean wait time (days)**	**89.4**	**104.2**	**108.9**	**121.3**	**120.7**	**221.6**
Pre‐operative oxford hip score						
0–4	−6.17	−4.81	−1.04	−18.19***	−8.98**	−46.47***
	(4.10)	(3.69)	(3.74)	(3.40)	(4.19)	(10.62)
5–8	−3.87**	−5.59***	−1.04	−4.21**	−9.15***	−30.25***
	(1.89)	(1.76)	(1.81)	(1.67)	(1.93)	(5.01)
9–12	−3.33**	−1.70	0.08	−2.86**	−4.33***	3.47
	(1.59)	(1.46)	(1.47)	(1.40)	(1.58)	(4.20)
13–16 (reference group)						
17–20	0.51	2.26*	4.95***	2.08	1.13	8.96**
	(1.46)	(1.35)	(1.38)	(1.31)	(1.49)	(4.22)
21–24	2.19	2.00	4.44***	3.72***	2.03	4.12
	(1.54)	(1.42)	(1.47)	(1.37)	(1.57)	(4.48)
25–28	4.66***	3.47**	6.03***	7.34***	3.83**	8.59
	(1.66)	(1.59)	(1.66)	(1.58)	(1.74)	(5.48)
29–32	2.41	5.24***	7.47***	8.94***	6.74***	40.71***
	(2.01)	(1.90)	(2.09)	(1.94)	(2.15)	(7.08)
33–48	7.93***	12.39***	12.08***	10.70***	12.87***	27.49***
	(2.27)	(2.20)	(2.49)	(2.33)	(2.57)	(7.84)

*Note:* Standard errors in parentheses. Coarsened exact k:m matching at patient level (k patients treated by private providers matched to m patients treated by public providers) on the following variables: Oxford Hip Score categories, sex, age categories, Index of Multiple Deprivation quintiles, the most common three primary diagnoses at admission (coxarthrosis unspecified, other primary coxarthrosis, polyarthrosis unspecified), the most common six secondary diagnoses of the Elixhauser morbidities recorded in the year preceding a hip replacement surgery (hypertension uncomplicated, obesity, chronic pulmonary disease, diabetes uncomplicated, cardiac arrhythmias, hypothyroidism). Marginal effects are presented for the Oxford Hip Score categories only. The model from which these marginal effects are derived is estimated using all patients from the matched samples and includes patient characteristics listed in Table 1, consultant fixed effects, and year and month dummy variables. Patient characteristics and consultant fixed effects are interacted with year dummy variables.

^*^ *p* < 0.1.

^**^ *p* < 0.05.

^***^ *p* < 0.01.

Prioritisation for patients with high pre‐operative health (above the reference group) appears comparable between public and private providers. Prioritisation differs instead for patients with low pre‐operative health. This is because patients with low pre‐operative health have shorter waiting times than the reference group within public providers but this is not the case for private providers where differences are limited and less systematic.^17^

While the above matching approaches produce matched samples that are balanced in terms of patient characteristics, waiting times for private providers remain significantly shorter compared to public providers even after matching. This motivates a second matching approach where we match each private provider with one public provider based on the average waiting time (coarsened) of each provider. This approach implicitly excludes private providers with short average waiting times that have no match with a public provider, and public providers with long average waiting times that have no match with a private provider. Supporting Information S1: Table A9 compares average waiting times between public and private providers before and after matching. The average waiting time for private providers increases after matching in the pre‐pandemic years (e.g., to 76 days in 2015/16 and 95 days in 2019/20) and to 205 days in 2020‐21.

Table 5 presents the results for the regressions on the matched samples. In the pre‐pandemic years, patients with better pre‐operative health (above the reference group) tend to wait longer in both public and private providers, but whether the effect is larger for public or private providers depends on the year. Patients with lower pre‐operative health (below the reference group) also wait less when treated by private providers while the effect for public providers is attenuated. These results suggest that wait prioritisation by public and private providers is more similar when restricting the comparison to hospitals with similar average waiting times.

**TABLE 5:** Inpatient waiting time prioritisation for public and private providers. Matched sample based on average waiting time by provider.

a. Private providers (independent sector)						
*N*. of observations	2015/16	2016/17	2017/18	2018/19	2019/20	2020/21
	(2493)	(2814)	(2024)	(3189)	(3084)	(973)
Pre‐operative oxford hip score						
0–4	−3.00	−15.12	3.30	−11.47	−23.14***	−53.97***
	(10.20)	(9.62)	(11.78)	(9.92)	(8.98)	(15.50)
5–8	−4.11	−3.07	−5.80	−2.43	−3.67	−42.96***
	(5.89)	(5.25)	(6.00)	(4.70)	(4.42)	(8.50)
9–12	−1.28	−0.58	0.05	−4.61	−2.67	−31.35***
	(4.74)	(4.28)	(4.84)	(4.02)	(3.78)	(7.48)
13–16 (reference group)						
17–20	−4.74	4.04	9.84**	−0.15	2.14	−11.64
	(4.22)	(4.06)	(4.58)	(3.65)	(3.66)	(7.54)
21–24	2.41	6.57	3.23	6.14*	6.11	−19.37**
	(4.41)	(4.21)	(5.02)	(3.70)	(3.76)	(8.20)
25–28	7.11	5.71	0.63	8.98**	8.35**	−15.21*
	(4.88)	(4.55)	(5.42)	(4.09)	(4.04)	(8.76)
29–32	3.48	1.29	−2.39	8.20	5.57	−7.99
	(5.26)	(5.61)	(6.59)	(5.25)	(4.89)	(10.35)
33–48	4.88	14.54**	17.75**	9.24	16.23***	7.96
	(6.14)	(6.01)	(7.46)	(5.71)	(5.33)	(12.35)

b. Public providers (NHS trusts)						
*N*. of observations	2015/16	2016/17	2017/18	2018/19	2019/20	2020/21
	(8934)	(11,151)	(9277)	(10,287)	(8504)	(2343)
Pre‐operative oxford hip score						
0–4	−3.61	−5.70**	−2.38	−5.74**	−5.45**	−13.00***
	(2.90)	(2.46)	(2.48)	(2.44)	(2.57)	(4.90)
5–8	−4.42**	−2.77*	−1.79	−1.82	−4.48**	−9.98***
	(1.87)	(1.64)	(1.71)	(1.68)	(1.84)	(3.20)
9–12	−1.80	−3.99***	−0.43	−0.39	−3.08*	−0.29
	(1.68)	(1.50)	(1.55)	(1.56)	(1.71)	(2.99)
13–16 (reference group)						
17–20	1.23	0.66	1.58	1.27	2.14	9.07***
	(1.66)	(1.47)	(1.56)	(1.56)	(1.74)	(3.26)
21–24	1.99	1.72	2.49	3.09*	3.61*	5.34
	(1.80)	(1.56)	(1.68)	(1.71)	(1.86)	(3.39)
25–28	5.51***	1.39	4.27**	2.53	2.40	23.62***
	(2.04)	(1.77)	(1.93)	(1.91)	(2.16)	(4.05)
29–32	4.77*	3.79*	8.90***	6.93***	3.60	28.80***
	(2.55)	(2.14)	(2.36)	(2.36)	(2.65)	(4.82)
33–48	7.48***	10.83***	11.62***	6.78**	5.87*	12.30**
	(2.85)	(2.39)	(2.76)	(2.76)	(3.02)	(5.52)

*Note:* Standard errors in parentheses. Coarsened exact k:k matching at provider level (k private providers matched to k public providers on their average inpatient waiting times). Marginal effects are presented for the Oxford Hip Score categories only. The model from which these marginal effects are derived is estimated at patient level using all patients from the matched samples and includes patient characteristics listed in Table 1, consultant fixed effects, and year and month dummy variables. Patient characteristics and consultant fixed effects are interacted with year dummy variables.

^*^ *p* < 0.1.

^**^ *p* < 0.05.

^***^ *p* < 0.01.

We compare outpatient wait prioritisation between public and private providers in Supporting Information S1: Table A10. This comparison is more tentative because the outpatient waiting time by a private provider can be preceded by outpatient appointments in a public provider, which is not measured in our database. The table suggests that there is some prioritisation in private providers, but it is even less pronounced relative to public providers.

### 5.5 Prioritisation on Different Dimensions of Health

We have so far focused on prioritisation based on the pre‐operative Oxford Hip Score. Supporting Information S1: Table A11 provides evidence of prioritisation on 12 individual domains on which the OHS is based. While each of the 12 questions has five possible answers, we create dummy variables that indicate the presence of serious or very serious health issues in each domain (see Supporting Information S1: Appendix C for details). The results show that it is mostly the pain dimensions that reduce inpatient waiting times. Patients experiencing severe pain (relative to moderate, mild, very mild or no pain) have shorter inpatient waiting times by 4 days in 2015/16–2019/20 and by 7 days in 2020/21. Mobility and ability to carry out daily activities do not generally affect waiting times.

### 5.6 Robustness Check

A substantial proportion of observations have missing values for inpatient waiting time, primarily due to missing or invalid dates for addition to the waiting list (see Supporting Information S1: Table A1). Since inpatient waiting time is central to our analysis, this raises the possibility of sample selection bias if missingness is systematically related to patient characteristics.

To assess the robustness of our results to this issue, we implement an inverse probability weighting (IPW) approach. Specifically, we estimate a logit model for the probability that inpatient waiting time is observed as a function of the full set of patient characteristics used in the main analysis. We then constructed stabilised inverse probability weights using the predicted probabilities from this model and re‐estimated our main regression using these weights.

The results reported in Supporting Information S1: Table A12 are highly consistent with the main results in Table 2. The relationship between pre‐operative health and inpatient waiting time remains qualitatively and quantitatively similar across all years. Patients with worse pre‐operative health continue to experience shorter waiting times relative to the reference group, while those with better health wait longer. The magnitude of these effects and their evolution over time, particularly the steeper gradient during the COVID‐19 period, are unchanged.

### 5.7 Simulation of Health Effects of Increased Prioritisation

Our findings provide some evidence of waiting time prioritisation across patients with differing pre‐operative health along the inpatient component of the patient pathway. In this section, we explore the health gains that could be attained by making prioritisation of patients more pronounced. Our simulation approach builds on the theoretical insights of Gravelle and Siciliani (2008b), who show that prioritisation based on observable patient characteristics improves welfare under certain conditions. In line with their model, we simulate the health gains from further redistributing waiting times from patients with high to low pre‐operative health (from low to high benefit from treatment). We follow their conceptual approach of comparing welfare before and after implementation of increased prioritisation, while maintaining the same total number of patients treated.

In Table 6, we summarise the information required to simulate the effect of increased prioritisation, and in Table 7 we simulate the effect of a reduction in waiting time for patients with low pre‐operative health at the expense of an increase in waiting times for patients with high pre‐operative health.

**TABLE 6:** Average HRQoL gain for patients with different pre‐operative health in 2019/20.

OHS category	Number of patients	Pre‐operative HRQoL (mean value)	Post‐operative HRQoL (mean value)	Inpatient waiting time	HRQoL gain (mean value)	Total health gain
0–4	901	−0.13	0.56	110	0.69	624
5–8	2592	−0.02	0.66	112	0.68	1763
9–12	3529	0.09	0.74	118	0.65	2278
13–16	3855	0.25	0.78	121	0.53	2026
17–20	3515	0.42	0.81	123	0.39	1373
21–24	2829	0.54	0.84	124	0.30	852
25–28	1864	0.60	0.86	125	0.26	478
29–32	1134	0.66	0.87	127	0.21	242
33–48	932	0.73	0.90	129	0.16	153
					**TOTAL**	**9789**

Abbreviations: HRQoL, health‐related quality of life; OHS, Oxford Hip Score.

**TABLE 7:** Simulation of health gain from additional prioritisation.

OHS category	Number of patients	Prioritised waiting time (−20%, +20%)	Change in waiting time from prioritisation	HRQoL gain per patient	Health (QALY) gain from prioritisation
0–4	901	88	−22	0.69	37.47
5–8	2592	90	−22	0.68	106.24
9–12	3529	94	−24	0.65	150.83
13–16	3855	121	0	0.53	0.00
17–20	3515	123	0	0.39	0.00
21–24	334	124	0	0.30	0.00
21–24	2495	149	25	0.30	−51.27
25–28	1864	150	25	0.26	−33.19
29–32	1134	152	25	0.21	−16.31
33–48	932	155	26	0.16	−10.62
				**TOTAL**	**183.14**

Abbreviations: HRQoL, health‐related quality of life; OHS, Oxford Hip Score.

Table 6 presents the mean pre‐ and post‐operative health‐related quality of life (HRQoL) in 2019/20 measured on the EQ‐5D scale with a score of 1 representing full health and zero representing death.^18^ Patients with lowest pre‐operative health have the largest health gain (0.69) and those with highest pre‐operative health have the lowest health gain (0.16), in line with the literature (e.g., Jenkins et al. 2013; Fordham et al. 2012; Price et al. 2020). We also show the mean waiting time for each OHS category using the mean waiting time in 2019/20 for the reference group (from Table 1) and the predicted waiting time for the other categories based on the regression results in Table 2. By multiplying the health gain with the total number of patients, we obtain the total health gain measured in quality‐adjusted life years (QALYs) for the patient group in the first year following surgery.

Table 7 presents a simulation of the potential health effects of additional prioritisation (see Supporting Information S1: Appendix E for details). We start by assuming that the demand is completely inelastic to waiting time and that waiting times do not affect health outcomes post‐surgery (though we relax these assumptions below). Define $h_{i\;}$ as the health gain of patients in group *i*, ${\;N}_{i\;}$ is the number of patients in group *i*, and $w_{i}^{A}$ and $w_{i}^{B}$ as waiting times (measured in years) before (period A) and after (period B) the introduction of the additional waiting‐time prioritisation. The total health gain for patients in group *i* is $T_{i\;}\left(w_{i}\right)={\;N}_{i\;}H_{i\;}\left(w_{i}\right)$, and the total health gain across all patients is $T\left(w\right)=\sum_{i=1}^{I}T_{i}\left(w_{i}\right)$. The effect of additional wait prioritisation on total health is equal to: $\Delta T=T\left(w^{B}\right)-T\left(w^{A}\right)=\sum_{i=1}^{I}N_{i}\;h_{i\;}\left(w_{B}^{i}-w_{A}^{i}\right)$.

We simulate the effect of a reduction in waiting time by 20% for patients in the three groups with highest health gain (7022 patients in total). All waiting time figures are rounded up to a day. We assume that hospitals have a fixed capacity and can treat a given number of patients. If some patients have a shorter wait and are admitted to hospital more quickly this needs to be offset by an increase in waiting time by other patients. This implies that the total waiting time across groups remains the same and therefore $\sum_{i=1}^{I}N_{i}\left(w_{i}^{B}-w_{i}^{A}\right)=0$. The total waiting time in the top three groups reduces by $\sum_{i=1}^{3}N_{i}\left(w_{i}^{B}-w_{i}^{A}\right)$, which is 442.58 years or 161,542 days.^19^ We therefore increase the waiting time by 20% for the three groups with lowest health gain and for 2495 out of the 2829 patients in the 21–24 OHS group (6425 patients in total). The total waiting time for the bottom four groups increases as a result by 161,557 days (with a very small 15 days difference due to rounding up of waiting times in days).^20^

The total health gain from this form of enhanced prioritisation is, using $\Delta T=\sum_{i=1}^{I}N_{i}\;h_{i\;}\left(w_{B}^{i}-w_{A}^{i}\right)$, equal to 183.14 QALYs. Patients with lowest pre‐operative health wait less and therefore benefit from the health gain more quickly. For example, for patients with a 0–4 OHS who have a shorter waiting time by 22 days, the total health gain for this group is equal to 37.47 QALYs (901 patients x 0.69 HRQoL x (22/365 years) = 37.47 QALYs). Patients with highest pre‐operative health instead experience a delay in health gain. For patients with 33–48 OHS who wait 26 days longer, the total health loss is equal to 10.62 QALYs (932 patients x 0.16 HRQoL x (26/365 years) = 10.62 QALYs). The health gains from the groups with lowest pre‐operative health is larger than the health loss from the groups with highest pre‐operative health, therefore leading to a net increase of 183.14 QALYs. This is equivalent to 1.9% of the QALY gain from all hip replacements provided in our sample in the first year. The gains from enhanced prioritisation are larger with even steeper wait prioritisation. For example, a larger reduction in waiting time of 40% for the patients with lowest pre‐operative health would increase aggregate health by 365.90 QALYs, which is about double the gain in the previous scenario.^21^

The analysis above relies on the assumptions that demand for surgery is completely inelastic and that waiting times do not affect health outcomes. We can relax these assumptions by assuming that health gain reduces with waiting time, ${\;h}_{i\;}\left(w_{i}\right)$ with $h_{i}^{\prime }\left(w_{i}\right)<0$, and that demand for surgery reduces with waitingtimes, ${\;N}_{i\;}\left(w_{i}\right)$ with $N_{i}^{\prime }\left(w_{i}\right)<0$. In this case, the effect on total health gain before and after the policy is $\Delta T=T\left(w^{B}\right)-T\left(w^{A}\right)$ where $T\left(w^{z}\right)=\sum_{i=1}^{I}N_{i}\left(w_{i}^{z}\right)\;{\;h}_{i\;}\left(w_{i}^{z}\right)\;\left[\sum_{t=0}^{T-1}\delta^{t}-w_{i}^{z}\right]$ and z={A,B} (see Supporting Information S1: Appendix E for details).

Suppose that waiting times do not affect health outcomes but that the demand is elastic. In line with existing evidence, we assume that the elasticity is equal to −0.1 so that a 10% increase in waiting time reduces demand by 1% (Martin and Smith 1999, 2003; Gravelle et al. 2003, Martin et al. 2007). We further assume that the elasticity does not vary by pre‐operative health group. Table 8 provides the results in this scenario using the same parameters provided in Table 7. A reduction in waiting times for patients with lowest pre‐operative health by 20% increases demand by 2%. Conversely, an increase in waiting times for patients with highest pre‐operative health reduces demand by 2%. Given that patients with worse health have a higher benefit from surgery, the overall health gain is now 185.96 QALYs relative to 183.14 identified when demand is completely inelastic (in Table 7). The difference appears quantitatively small.

**TABLE 8:** Simulation of health gain from additional prioritisation with elastic demand.

OHS category	Number of patients	Prioritised waiting time (−20%, +20%)	Change in waiting time from prioritisation	HRQoL gain per patient	Health (QALY) gain from prioritisation
0–4	919	88	−22	0.69	38.22
5–8	2644	90	−22	0.68	108.36
9–12	3600	94	−24	0.65	153.85
13–16	3855	121	0	0.53	0.00
17–20	3515	123	0	0.39	0.00
21–24	69	124	0	0.30	0.00
21–24	2703	149	25	0.30	−55.55
25–28	1827	150	25	0.26	−32.53
29–32	1111	152	25	0.21	−15.98
33–48	913	155	26	0.16	−10.41
				TOTAL	185.96

*Note:* Which measures pre‐operative health for different OHS categories.

Abbreviations: HRQoL, health‐related quality of life; OHS, Oxford Hip Score.

Last, we assume that waiting times reduce health outcomes, but demand is completely inelastic. In line with Nikolova et al. (2016) we assume that an increase in waiting time by a week reduces post‐operative health by 0.05% (and equal to 0.00038 points on the HRQoL scale given an average post‐operative health‐related quality of life of 0.76).^22^ The results are presented in Supporting Information S1: Table A15. Patients with lower pre‐operative health have larger health gain per patient from reduced wait, while patients with better pre‐operative health have smaller health gain because of the higher wait. The findings are again similar to those reported in Table 7 with an overall health gain of 183.19 QALYs (rather than 183.14).^23^

This simple approach makes several assumptions. We do not model the length of the health gain from treatment and any subsequent differences according to patients being treated earlier as a result of prioritisation. For example, our approach assumes that patients receiving treatment earlier do not suffer an earlier decline in their hip function as the implant ages. While this is a limitation, it is unlikely to make a large difference to our results given that the health gain from a hip replacement only declines slowly over time (Fordham et al. 2012) and differences in health gain far into the future are likely to be less important due to discounting. We also do not allow for any heterogeneity in patients' health gain from treatment (apart from pre‐operative OHS), and assume each patient has the same health gain associated with their OHS category.

## 6 Discussion and Conclusion

This study provides evidence of waiting time prioritisation based on pre‐operative health for hip replacement patients in England. Between 2015/16 and 2019/20, there was a difference of about 15–21 days in inpatient waiting time between those with the lowest and highest pre‐operative health prior to surgery. This suggests that doctors prioritise patients even in the absence of explicit guidelines to reduce the disutility from waiting (Gutacker et al. 2016; Gravelle and Siciliani 2008b).

This mechanism is further corroborated by how prioritisation changed over time both before and after COVID‐19. Inpatient wait prioritisation became steeper when waiting times were longer. In 2018/19, when waiting times were on average 120 days, the difference between the lowest and highest pre‐operative health was 21 days, while in 2015/16 when waiting times were on average 90 days the difference between the two groups was only 13 days. This suggests that as the waiting time increases, doctors further differentiate between patients with differing levels of health by giving priority to patients who are less mobile and have more pain in the joint.

The onset of the COVID‐19 pandemic generated a dramatic increase in average waiting times to 219 days in 2020/21 following the suspension of elective care and subsequent reduction in volume. During this period we document a further increase in prioritisation with a difference of up to 47 days in waiting time between those in high and lowest pre‐operative health. In addition to informal prioritisation, the steeper gradient may also reflect the introduction of guidance by the surgical specialty association recommending prioritising patients for whom long delays could affect health outcomes (Federation of Specialty Surgical Associations 2021).^24^

We show that outpatient waiting times, between the referral of a general practitioner or other sources and the specialist visit, comprise a substantial proportion of total time waited (from referral to surgery). Therefore, outpatient waiting times constitute another opportunity to prioritise along the patient pathway. In contrast to the findings on inpatient wait prioritisation, we find more limited evidence of outpatient waiting time prioritisation. Patients with highest pre‐operative health wait at most 13 days longer than patients with middle pre‐operative health, for a given number of appointments, but patients with lowest pre‐operative health generally wait the same as the middle group. Therefore, prioritisation is less pronounced than for inpatient waiting time.

The main mechanism at work behind the outpatient prioritisation gradient is that patients with better pre‐operative health have more outpatient appointments and this contributes to a longer overall outpatient waiting time. When we instead look only at patients who have a single outpatient appointment after being referred by a GP, we find no evidence of outpatient waiting time prioritisation by pre‐operative health. One interpretation is that multiple outpatient appointments are not simply a source of delay, but part of the specialist assessment and selection process, particularly for patients with better pre‐operative health for whom the appropriateness of surgery may be less clear. This suggests that part of the observed outpatient wait gradient may reflect extended decision‐making about whether and when to proceed to surgery, rather than explicit waiting time prioritisation.

There may be scope for developing policies that encourage GPs to indicate the degree of priority in their referral to the specialist. There have been initiatives across countries to improve the coordination between primary and secondary care to ensure appropriate and timely referrals, for example in Canada, Italy and Finland (OECD 2020). Some evidence from Italy suggests that GPs and specialists may however disagree in the assessment of patient priority. GPs tend to assign a higher priority than specialists though agreement between GPs and specialists can be facilitated by electronic referral management tools (Mariotti et al. 2022).

Driven by a stronger profit motive, private providers could have an incentive to dampen wait prioritisation. The same profit motive could induce private providers to treat less complex patients (Street et al. 2010) and have a shorter waiting time to attract patients and increase demand. We show that after controlling for casemix and average waiting times, wait prioritisation is similar between public and private providers. Although this is reassuring in terms of equity implications, private providers have much shorter waiting times, and this can create inequalities in waiting times based on the geographical distribution of private providers.

Our simulations illustrate that there is scope to introduce policies that enhance waiting time prioritisation. In line with the theory (Gravelle and Siciliani 2008b), prioritisation policies that redistribute waiting times from patients with low pre‐operative to patients with high pre‐operative health can generate health gains. Our analysis is able to quantify such gains. We consider a (realistic) increase in waiting times by a further 20% for patients with the best pre‐operative health (who have the smallest health gains) and simultaneous similar reduction in waiting times for patients with the worse pre‐operative health (who have the largest health gains). We show that such intervention would increase overall health by 1.9% of the first year of health gain experienced by hip replacement patients post procedure. These estimates are robust to different assumptions regarding the demand elasticity to waiting times, and the extent to which waiting times affect patient ability to benefit from health care.

Such health improvements could be achieved without additional cost to the health budget, as it effectively redistributes the disutility of waiting across different patients. The simulation however assumes waiting times can be reallocated flexibly across patients with different pre‐operative health. In practice, such reallocation could face implementation challenges. The main practical constraint would be establishing systems to measure pre‐operative health status earlier in the referral pathway, rather than just before surgery as is current practice. Any prioritisation system based on self‐reported health measures would also need safeguards against potential gaming, where patients might strategically overstate symptoms to secure shorter waiting times.

Last, our analysis shows that patients living in more deprived areas face higher inpatient waits, but the effect is relatively modest in the pre‐COVID period, at most 4 days for patients in the most deprived quintile. However, these inequalities are much more pronounced in the COVID‐19 year, with patients in the most deprived quintile waiting 20 days longer than those in the least deprived one. This suggests that inequalities are likely to arise when waiting times increase. We did not find evidence of inequalities in outpatient waiting times by income deprivation.

Overall, our analysis suggests that there is scope for improving prioritisation of the patients on the list along the patient pathway both in the inpatient and the outpatient component. This is especially important when waiting times are long, as in England in recent years and in particular post‐pandemic. Waiting time prioritisation can become more salient through further informal or formal guidance (Federation of Specialty Surgical Associations 2021), and a widespread debate about how to prioritise patients to improve the equity of outcomes at a local level (Shenoy 2023). Policymakers at a national level could further develop robust frameworks and criteria for waiting time prioritisation to ensure equity and efficiency goals are being achieved.

## Conflicts of Interest

The authors declare no conflicts of interest.

## Supporting information


Supporting Information S1

## Data Availability

The data that support the findings of this study are available from NHS England. Restrictions apply to the availability of these data, which were used under license for this study.

## Endnotes

^1^
Marques et al. (2014) uses data from the UK from 2006 to 2009 on both inpatient and outpatient waiting times (which they label the “official” and “work‐up” waiting time). They find very high outpatient waiting times (mean 429–532 days compared to 88–157 days for inpatient waits) and that patients living in the least deprived 20% of areas experience shorter outpatient waits but no difference in inpatient waiting time.

^2^
For hip replacement, Laudicella et al. (2012) find that in 2001 patients in the fourth and fifth most income‐deprived quintile waited about 7% longer than patients in the least deprived quintile, though it reduced by 2010 (Moscelli et al. 2023). Also for hip replacement in 2000‐03, Monstad et al. (2014) find that in Norway men in the highest income group waited 14% less relative to those with lowest income. Women with the highest education waited 7% less relative to those with lowest education. Simonsen et al. (2020) find a socio‐economic waiting time gradient for hernia (8–17 days) and cataract surgery (4–5 days) using data from Denmark. For coronary bypass in England, Moscelli, Siciliani, et al. (2018) found up to 35% (43 days) difference in waiting time between patients in the most and least deprived quintiles, and that only up to 12% of these waiting time inequalities can be attributed to patients' choices of hospital and type of treatment (heart bypass vs. stent). Johar et al. (2013) find that in Australia (New South Wales) more deprived patients in need of elective treatment wait 16%–24% longer. Sharma et al. (2013) find that in Australia (Victoria), patients living in areas in the highest income decile wait overall 12% less.

^3^
The procedure codes for the various types of hip replacements are defined in the PROMs guide (NHS Digital 2017). They are also listed in Supporting Information S1: Appendix B.

^4^
Completion of the pre‐operative PROMs questionnaire is voluntary for the patient and administered by the hospital between the last outpatient appointment and the surgery. The post‐operative questionnaire is posted to patients at least 6 months after surgery. The PROMs data are used to determine the health gain and impact on symptoms and functional status. We only use questionnaires on health prior to surgery.

^5^
Not all PROM questionnaires can be linked to HES. Unlinked questionnaires occur because: (a) patient details on the questionnaire are poorly completed, (b) clinical coding in the episode may be missing or poorly completed failing to identify the episode as a hip replacement and making it ineligible for matching, (c) there are provider mapping issues, (d) patient died before the operation, (e) patient cancelled the operation. Some patients had more than one hip replacement in the same year and therefore submitted more than one questionnaire. We only use records related to the first questionnaire submitted by the patient.

^6^
Patients with very long recorded waiting times are likely where waiting times do not represent resource constraints and may be due to misrecording of the date when the patient is added to the list, or other factors.

^7^
We exclude privately‐funded patients treated in NHS and private hospitals.

^8^
There are some outpatient appointments, for which the consultant's specialty is not “Orthopaedics” but matches the specialty of the consultant responsible for the inpatient admission. These appointments are also included.

^9^
As an example, consider the case of a hypothetical patient who was added to the hip replacement waiting list on 1st July 2017. For this individual, we identify two spells: the first outpatient spell consists of a single appointment that has a referral request date on the 15th May 2017 and was attended on the 20th June 2017; the second spell consists of three appointments that had the same referral request date of 1st February 2017 and they were attended on 1st April, 15th May and 1st July of the same year.

^10^
Specifically, we include dummies for the 10 most frequent primary diagnoses (using the International Classification of Diseases (ICD) version 10 codes) and a dummy indicating any other primary diagnosis. The most common primary diagnoses are different types of coxarthrosis. The Elixhauser Comorbidity Index is a measure of patient burden of disease, which is based on 31 chronic conditions (comorbidities) present in a patient (e.g., diabetes, heart failure, or liver disease). As well as the primary diagnosis for the admission, we create dummy variables indicating the presence of each of the conditions of the Elixhauser index recorded in any of the admissions in the previous year (including as secondary diagnoses in the current admission). This results in 30 dummy variables, which allow us to control for the complexity of a patients' health status. To capture symptom duration we include information about whether patients experienced problems with their hip for less than a year, 1–5 years, 6–10 years, and more than 10 years.

^11^
As robustness checks, we estimate models with a logged dependent variable and count data models, such as Poisson or negative binomial (Gutacker et al. 2016; Siciliani and Verzulli 2009).

^12^
One possible explanation is that some patients may have complications or experience other health problems that require postponing the surgery. Others may face challenges in their personal life that may make it difficult to proceed with surgery. Some of the less complex patients may also experience long delays to ensure that surgery is appropriate for them. The finding that the median waiting times is below the mean is not specific to hip replacement but it is very common across many procedures within the English NHS and across health systems (OECD 2013, 2020).

^13^
Although the waiting time is technically a count, it takes on a wide range of values and behaves more like a continuous variable in practice. This makes the linear model a suitable choice, as it avoids overfitting or imposing strong distributional assumptions. We present results from a Poisson regression model in the appendix (Supporting Information S1: Table A13). These results are broadly consistent with those from the linear model.

^14^
Patients in the 18–44 age group wait 7–13 days longer than patients in the 65–74 age group in 2015/16–2017/18.

^15^
The magnitude of the effect is much larger in 2020/21: waiting time for patients with one emergency admission (relative to no admission) is 4 days shorter in 2018/19 but 22 days shorter in 2020/21. Those with more than one emergency admission had 10 days shorter waiting times in 2018/19 but 54 days shorter in 2020/21. Patients with previous emergency admissions were prioritised more heavily during the pandemic.

^16^
In 2015/16 patients who experienced symptoms for more than 10 years waited 5 days longer than those whose symptoms had lasted only 1–5 years (the reference group), who waited nearly 12 days longer than those experiencing symptoms only in the past year. As with the gradient on pre‐operative health, these effects grow over time. By 2019, patients with the longest symptom duration wait 9 days longer than those with a symptom duration of 1–5 years, and those with the most recent symptom onset wait 21 days less than the base category. The beginning of the pandemic sees even larger effects with patients with the most recent symptom onset waiting 85 days less than those in the base category in 2020‐21.

^17^
As a robustness check, we also used samples with a 1:1 coarsened exact matching on patient characteristics. The results are qualitatively similar.

^18^
The HRQoL scores are for NHS providers and from the PROMs dataset. They measure the EQ‐5D component of the PROMS surveys with the three levels of each of the five dimensions of health valued according to the results from Dolan (1997) with a score of 1 representing full health and zero representing death.

^19^
The total waiting time reduces by 901*22 + 2592*22 + 3529*24 = 161,542 days.

^20^
The total waiting time increase is given by 932*26 + 1134*25 + 1864*25 + 2495*25 = 161,557 days.

^21^
Supporting Information S1: Table A14 in Appendix E simulates a larger reduction in waiting time of 40% for the bottom three OHS categories and an increase in waiting time of 40% for the top three OHS categories. The total waiting time in the top three groups reduces by 322,147 days and this is compensated by an increase in the total waiting time for the bottom four groups by 322,148 days. The net increase in health from this change in prioritisation is now given by 365.90 QALYs.

^22^
For example, for patients in group 0–4, we assume that each patient loses 0.76*0.0005*(22/7) = 0.002787 on the HRQoL scale.

^23^
We do not model the probability of dying while waiting. Our study focuses on hip replacements for elective patients for whom the mortality risk is negligible.

^24^
In line with Nikolova et al. (2016), we also find that symptom duration is associated with longer waiting times, with patients with recent symptoms having much shorter waiting time. This may capture an association between symptom duration and perceived severity, with evidence suggesting that short symptom duration prior to surgery is associated with lower pre‐operative health and a larger health gain (Lau et al. 2020).
