# Supplementary material for: Waiting Time Prioritisation for Public and Private Providers: Evidence From Hip Replacement Surgery
Source: Health Econ. 2026 Aug 8;35(11):1744–63. doi: 10.1002/hec.70140 (PMC13632919; doi:10.1002/hec.70140)
Supplement: Supplementary file 1 [file HEC-35-1744-s001.docx]

**Appendix A.**

**Table A1. Sample selection**

|  | 2015/16 | 2016/17 | 2017/18 | 2018/19 | 2019/20 | | 2020/21 |  |
| --- | --- | --- | --- | --- | --- | --- | --- | --- |
| Hip Replacements in HES* | 79,367 | 82,432 | 79,038 | 83,157 | 79,554 | | 31,940 |  |
| bilateral hip replacements | 563 | 564 | 517 | 562 | 518 | | 275 |  |
| revisions | 6,323 | 5,998 | 5,535 | 5,485 | 4,883 | | 2,120 |  |
| Hip Replacements  (excl. bilateral and revisions) | 72,481 | 75,870 | 72,986 | 77,110 | 74,153 | | 29,545 |  |
| With pre-operative responses** | 45,587 | 50,935 | 46,550 | 50,879 | 44,131 | | 11,249 |  |
| Pre-operative response rate*** | (63%) | (67%) | (64%) | (66%) | (60%) | | (38%) |  |
| Drop observations: |  |  |  |  |  |  | | |
| response not the first in year | 782 | 919 | 774 | 897 | 650 | 99 | | |
| missing sex | 4 | 5 | 0 | 3 | 5 | 0 | | |
| missing age | 0 | 0 | 1 | 1 | 1 | 0 | | |
| age<18 | 8 | 8 | 12 | 14 | 10 | 4 | | |
| missing rurality | 137 | 61 | 58 | 51 | 41 | 17 | | |
| missing IP waiting time | 5,909 | 5,919 | 3,804 | 3,668 | 3,781 | 1,506 | | |
| missing IMD | 306 | 300 | 382 | 443 | 432 | 91 | | |
| missing symptom duration | 493 | 344 | 291 | 395 | 446 | 72 | | |
| independent sector providers | 9,807 | 11,450 | 11,978 | 15,023 | 14,063 | 3,349 | | |
| inpatient waiting > 3 std dev. | 413 | 366 | 245 | 320 | 236 | 18 | | |
| **Inpatient sample** | 27,728 | 31,563 | 29,005 | 30,064 | 24,466 | 6,093 | | |
| Linked with outpatient | 21,873 | 24,795 | 22,848 | 24,002 | 19,574 | 4,652 | | |
|  | (79%) | (79%) | (79%) | (80%) | (80%) | (76%) | | |
| Drop observations with |  |  |  |  |  |  | | |
| > 10 appointments in spell | 109 | 129 | 129 | 145 | 112 | 27 | | |
| outpatient waiting time > 3 std dev. | 572 | 607 | 553 | 624 | 506 | 121 | | |
| **Regression sample** | 21,192 | 24,059 | 22,166 | 23,233 | 18,956 | 4,414 | | |
| **Restricted sample****** | 11,171 | 12,531 | 11,097 | 11,265 | 9,204 | 1,891 | | |

**Notes**: *Restricted to elective admissions.

**Questionnaires that are not linked with HES are excluded. Unlinked questionnaires occur because: a) patient details on the questionnaire are poorly completed, b) clinical coding in the episode may be missing or poorly completed failing to identify the episode as a hip replacement and making it ineligible for matching, c) there are provider mapping issues, d) patient died before the operation, e) patient cancelled the operation.

***The participation rate reported in the NHS Digital PROMS publications is defined as the number of pre-operative questionnaires (linked and unlinked) received per 100 PROMS-eligible hospital procedures and exceeds 80% during the period 2015/16-2019/20. The pre-operative response rate we report here is different from the participation rate in that it excludes unlinked pre-operative questionnaires. As a result, the pre-operative response rate is lower.

****Sample includes only GP referrals and single outpatient appointment spells.

**Table A2. Sample representativeness**

|  |  | 2019/20 | |  | 2020/21 | |
| --- | --- | --- | --- | --- | --- | --- |
|  | Original sample* (N=67,946) | Non respondents** (N=20,900) | Final sample*** (N=18,956) | Original sample (25,820) | Non respondents (N=10,831) | Final sample (N=4,414) |
| **Inpatient waiting time (mean)** | 109.883 | 131.871 | 120.798 | 188.192 | 226.850 | 219.467 |
| **Male** | 0.403 | 0.407 | 0.400 | 0.403 | 0.404 | 0.390 |
| **Age group** |  |  |  |  |  |  |
| 18-44 | 0.027 | 0.031 | 0.028 | 0.032 | 0.034 | 0.039 |
| 45-54 | 0.085 | 0.082 | 0.082 | 0.096 | 0.090 | 0.093 |
| 55-64 | 0.216 | 0.190 | 0.215 | 0.233 | 0.217 | 0.220 |
| 65-74 (reference group) | 0.354 | 0.332 | 0.346 | 0.340 | 0.323 | 0.355 |
| 75-84 | 0.267 | 0.293 | 0.276 | 0.253 | 0.276 | 0.249 |
| >=85 | 0.052 | 0.072 | 0.052 | 0.046 | 0.059 | 0.045 |
| **Ethnicity** |  |  |  |  |  |  |
| White (reference group) | 0.805 | 0.844 | 0.878 | 0.745 | 0.846 | 0.849 |
| Mixed | 0.002 | 0.002 | 0.002 | 0.003 | 0.004 | 0.001 |
| Asian | 0.006 | 0.008 | 0.005 | 0.006 | 0.007 | 0.009 |
| Black | 0.007 | 0.010 | 0.007 | 0.007 | 0.008 | 0.007 |
| Other ethnicity | 0.004 | 0.006 | 0.005 | 0.004 | 0.006 | 0.003 |
| Missing Ethnicity | 0.176 | 0.130 | 0.104 | 0.235 | 0.130 | 0.130 |
| **Rural (reference group: urban)** | 0.280 | 0.267 | 0.274 | 0.281 | 0.271 | 0.294 |
| **Emergency admissions** |  |  |  |  |  |  |
| 0 (reference group) | 0.907 | 0.881 | 0.903 | 0.912 | 0.889 | 0.922 |
| 1 | 0.070 | 0.087 | 0.076 | 0.064 | 0.086 | 0.059 |
| >=2 | 0.023 | 0.031 | 0.022 | 0.024 | 0.025 | 0.018 |
| **Primary diagnosis** |  |  |  |  |  |  |
| Coxarthr., unsp. (ref. group) | 0.783 | 0.753 | 0.773 | 0.778 | 0.745 | 0.750 |
| Other primary coxarthrosis | 0.064 | 0.063 | 0.084 | 0.053 | 0.043 | 0.101 |
| Polyarthrosis, unspecified | 0.078 | 0.078 | 0.070 | 0.075 | 0.076 | 0.061 |
| Osteonecrosis, unspecified | 0.009 | 0.012 | 0.009 | 0.012 | 0.015 | 0.012 |
| Primary coxarthrosis, bilateral | 0.006 | 0.007 | 0.007 | 0.004 | 0.005 | 0.007 |
| **Secondary diagnosis** |  |  |  |  |  |  |
| Hypertension, uncomplicated | 0.475 | 0.512 | 0.489 | 0.441 | 0.464 | 0.440 |
| Obesity | 0.266 | 0.268 | 0.256 | 0.244 | 0.244 | 0.249 |
| Chronic pulmonary disease | 0.171 | 0.188 | 0.179 | 0.159 | 0.170 | 0.169 |
| Diabetes, uncomplicated | 0.106 | 0.128 | 0.111 | 0.092 | 0.106 | 0.100 |
| Cardiac arrhythmias | 0.108 | 0.138 | 0.109 | 0.097 | 0.124 | 0.094 |
| Hypothyroidism | 0.095 | 0.099 | 0.101 | 0.088 | 0.089 | 0.089 |
| Depression | 0.093 | 0.095 | 0.086 | 0.095 | 0.094 | 0.096 |
| Renal failure | 0.084 | 0.098 | 0.085 | 0.071 | 0.084 | 0.071 |
| Rheumatoid arthritis | 0.063 | 0.075 | 0.066 | 0.062 | 0.068 | 0.073 |
| Valvular disease | 0.039 | 0.055 | 0.042 | 0.030 | 0.042 | 0.029 |
| **IMD quintile** |  |  |  |  |  |  |
| 1 (ref. group - least deprived) | 0.237 | 0.217 | 0.229 | 0.243 | 0.221 | 0.250 |
| 2 | 0.237 | 0.223 | 0.228 | 0.245 | 0.232 | 0.245 |
| 3 | 0.216 | 0.225 | 0.213 | 0.220 | 0.225 | 0.227 |
| 4 | 0.177 | 0.187 | 0.184 | 0.171 | 0.184 | 0.167 |
| 5 (most deprived) | 0.133 | 0.147 | 0.146 | 0.121 | 0.138 | 0.111 |

**Notes.**

* Sample of patients undergone a hip replacement (excluding bilateral operations and revisions) in either NHS or private hospitals, excluding missing values on age, sex, rurality, inpatient waiting times, and symptom duration.

** Sample of patients undergone a hip replacement (excluding bilateral operations and revisions) in NHS hospitals who did not provide pre-operative responses excluding missing values on age, sex, rurality, inpatient waiting times, and symptom duration.

*** Sample of patients undergone a hip replacement (excluding bilateral operations, revisions) in NHS hospitals who provided pre-operative responses, and were linked to outpatient appointments, excluding missing values on age, sex, rurality, inpatient waiting times, and symptom duration (Regression sample in Table A1).

**Table A3. Comparison between non-respondents and final sample: 2015/16 to 2017/18**

|  | 2015/16 | | 2016/17 | | 2017/18 | |
| --- | --- | --- | --- | --- | --- | --- |
|  | Non respondents* (N=18,757) | Final sample** (N=21,192) | Non respondents* (N=15,839) | Final sample** (N=24,059) | Non respondents* (N=15,980) | Final sample** (N=22,166) |
| **Inpatient waiting time (mean)** | 96.173 | 90.120 | 111.695 | 103.846 | 120.177 | 109.362 |
| **Male** | 0.399 | 0.404 | 0.404 | 0.402 | 0.408 | 0.402 |
| **Age group** |  |  |  |  |  |  |
| 18-44 | 0.035 | 0.031 | 0.033 | 0.030 | 0.033 | 0.030 |
| 45-54 | 0.083 | 0.085 | 0.083 | 0.087 | 0.083 | 0.090 |
| 55-64 | 0.196 | 0.205 | 0.191 | 0.203 | 0.193 | 0.200 |
| 65-74 (reference group) | 0.329 | 0.353 | 0.333 | 0.357 | 0.336 | 0.360 |
| 75-84 | 0.291 | 0.277 | 0.289 | 0.270 | 0.291 | 0.267 |
| >=85 | 0.065 | 0.048 | 0.070 | 0.053 | 0.064 | 0.052 |
| **Ethnicity** |  |  |  |  |  |  |
| White (reference group) | 0.885 | 0.892 | 0.881 | 0.890 | 0.865 | 0.882 |
| Mixed | 0.002 | 0.002 | 0.003 | 0.002 | 0.003 | 0.002 |
| Asian | 0.007 | 0.005 | 0.007 | 0.005 | 0.007 | 0.006 |
| Black | 0.007 | 0.007 | 0.007 | 0.006 | 0.008 | 0.007 |
| Other ethnicity | 0.006 | 0.004 | 0.004 | 0.005 | 0.005 | 0.005 |
| Missing Ethnicity | 0.093 | 0.090 | 0.098 | 0.092 | 0.113 | 0.099 |
| **Rural (reference group: urban)** | 0.243 | 0.253 | 0.260 | 0.270 | 0.263 | 0.274 |
| **Emergency admissions** |  |  |  |  |  |  |
| 0 (reference group) | 0.889 | 0.919 | 0.888 | 0.913 | 0.886 | 0.910 |
| 1 | 0.083 | 0.064 | 0.084 | 0.068 | 0.086 | 0.072 |
| >=2 | 0.028 | 0.017 | 0.028 | 0.019 | 0.028 | 0.018 |
| **Primary diagnosis** |  |  |  |  |  |  |
| Coxarthr., unsp. (ref. group) | 0.725 | 0.740 | 0.741 | 0.755 | 0.758 | 0.768 |
| Other primary coxarthrosis | 0.088 | 0.107 | 0.073 | 0.095 | 0.061 | 0.079 |
| Polyarthrosis, unspecified | 0.063 | 0.061 | 0.063 | 0.067 | 0.073 | 0.073 |
| Osteonecrosis, unspecified | 0.014 | 0.010 | 0.013 | 0.011 | 0.012 | 0.011 |
| Primary coxarthrosis, bilateral | 0.013 | 0.017 | 0.010 | 0.010 | 0.005 | 0.007 |
| **Secondary diagnosis** |  |  |  |  |  |  |
| Hypertension, uncomplicated | 0.496 | 0.482 | 0.507 | 0.481 | 0.506 | 0.493 |
| Obesity | 0.157 | 0.154 | 0.178 | 0.173 | 0.208 | 0.200 |
| Chronic pulmonary disease | 0.172 | 0.162 | 0.176 | 0.167 | 0.183 | 0.172 |
| Diabetes, uncomplicated | 0.118 | 0.109 | 0.122 | 0.110 | 0.127 | 0.111 |
| Cardiac arrhythmias | 0.117 | 0.092 | 0.122 | 0.097 | 0.126 | 0.107 |
| Hypothyroidism | 0.086 | 0.080 | 0.087 | 0.088 | 0.095 | 0.089 |
| Depression | 0.062 | 0.060 | 0.069 | 0.066 | 0.075 | 0.077 |
| Renal failure | 0.079 | 0.068 | 0.090 | 0.072 | 0.092 | 0.077 |
| Rheumatoid arthritis | 0.059 | 0.055 | 0.059 | 0.061 | 0.070 | 0.065 |
| Valvular disease | 0.041 | 0.028 | 0.043 | 0.032 | 0.041 | 0.036 |
| **Index of Multiple Deprivation (IMD) quintile** |  |  |  |  |  |  |
| 1 (ref. group - least deprived) | 0.215 | 0.221 | 0.215 | 0.225 | 0.216 | 0.227 |
| 2 | 0.227 | 0.227 | 0.224 | 0.233 | 0.224 | 0.232 |
| 3 | 0.215 | 0.211 | 0.217 | 0.214 | 0.227 | 0.216 |
| 4 | 0.189 | 0.185 | 0.192 | 0.180 | 0.183 | 0.182 |
| 5 (most deprived) | 0.154 | 0.155 | 0.152 | 0.148 | 0.150 | 0.144 |

**Notes.**

* Sample of patients undergone a hip replacement (excluding bilateral operations and revisions) in NHS hospitals who did not provide pre-operative responses excluding missing values on age, sex, rurality, inpatient waiting times, and symptom duration.

** Sample of patients undergone a hip replacement (excluding bilateral operations, revisions) in NHS hospitals who provided pre-operative responses, and were linked to outpatient appointments, excluding missing values on age, sex, rurality, inpatient waiting times, and symptom duration (Regression sample in Table A1).

**Table A3 (continued). Comparison between non-respondents and final sample: 2018/19 to 2020/21**

|  | 2018/19 | | 2019/20 | | 2020/21 | |
| --- | --- | --- | --- | --- | --- | --- |
|  | Non respondents* (N=17,875) | Final sample** (N=23,233) | Non respondents* (N=20,900) | Final sample** (N=18,956) | Non respondents* (N=10,831) | Final sample** (N=4,414) |
| **Inpatient waiting time (mean)** | 134.404 | 120.487 | 131.871 | 120.798 | 226.850 | 219.467 |
| **Male** | 0.400 | 0.407 | 0.407 | 0.400 | 0.404 | 0.390 |
| **Age group** |  |  |  |  |  |  |
| 18-44 | 0.030 | 0.029 | 0.031 | 0.028 | 0.034 | 0.039 |
| 45-54 | 0.081 | 0.083 | 0.082 | 0.082 | 0.090 | 0.093 |
| 55-64 | 0.189 | 0.205 | 0.190 | 0.215 | 0.217 | 0.220 |
| 65-74 (reference group) | 0.336 | 0.353 | 0.332 | 0.346 | 0.323 | 0.355 |
| 75-84 | 0.293 | 0.277 | 0.293 | 0.276 | 0.276 | 0.249 |
| >=85 | 0.071 | 0.053 | 0.072 | 0.052 | 0.059 | 0.045 |
| **Ethnicity** |  |  |  |  |  |  |
| White (reference group) | 0.854 | 0.881 | 0.844 | 0.878 | 0.846 | 0.849 |
| Mixed | 0.003 | 0.002 | 0.002 | 0.002 | 0.004 | 0.001 |
| Asian | 0.007 | 0.005 | 0.008 | 0.005 | 0.007 | 0.009 |
| Black | 0.007 | 0.007 | 0.010 | 0.007 | 0.008 | 0.007 |
| Other ethnicity | 0.007 | 0.004 | 0.006 | 0.005 | 0.006 | 0.003 |
| Missing Ethnicity | 0.122 | 0.101 | 0.130 | 0.104 | 0.130 | 0.130 |
| **Rural (reference group: urban)** | 0.277 | 0.278 | 0.267 | 0.274 | 0.271 | 0.294 |
| **Emergency admissions** |  |  |  |  |  |  |
| 0 (reference group) | 0.880 | 0.907 | 0.881 | 0.903 | 0.889 | 0.922 |
| 1 | 0.090 | 0.075 | 0.087 | 0.076 | 0.086 | 0.059 |
| >=2 | 0.030 | 0.018 | 0.031 | 0.022 | 0.025 | 0.018 |
| **Primary diagnosis** |  |  |  |  |  |  |
| Coxarthr., unsp. (ref. group) | 0.751 | 0.766 | 0.753 | 0.773 | 0.745 | 0.750 |
| Other primary coxarthrosis | 0.060 | 0.084 | 0.063 | 0.084 | 0.043 | 0.101 |
| Polyarthrosis, unspecified | 0.077 | 0.073 | 0.078 | 0.070 | 0.076 | 0.061 |
| Osteonecrosis, unspecified | 0.013 | 0.011 | 0.012 | 0.009 | 0.015 | 0.012 |
| Primary coxarthrosis, bilateral | 0.006 | 0.007 | 0.007 | 0.007 | 0.005 | 0.007 |
| **Secondary diagnosis** |  |  |  |  |  |  |
| Hypertension, uncomplicated | 0.517 | 0.492 | 0.512 | 0.489 | 0.464 | 0.440 |
| Obesity | 0.244 | 0.227 | 0.268 | 0.256 | 0.244 | 0.249 |
| Chronic pulmonary disease | 0.194 | 0.178 | 0.188 | 0.179 | 0.170 | 0.169 |
| Diabetes, uncomplicated | 0.126 | 0.112 | 0.128 | 0.111 | 0.106 | 0.100 |
| Cardiac arrhythmias | 0.136 | 0.109 | 0.138 | 0.109 | 0.124 | 0.094 |
| Hypothyroidism | 0.098 | 0.094 | 0.099 | 0.101 | 0.089 | 0.089 |
| Depression | 0.086 | 0.081 | 0.095 | 0.086 | 0.094 | 0.096 |
| Renal failure | 0.094 | 0.079 | 0.098 | 0.085 | 0.084 | 0.071 |
| Rheumatoid arthritis | 0.074 | 0.065 | 0.075 | 0.066 | 0.068 | 0.073 |
| Valvular disease | 0.051 | 0.038 | 0.055 | 0.042 | 0.042 | 0.029 |
| **Index of Multiple Deprivation (IMD) quintile** |  |  |  |  |  |  |
| 1 (ref. group - least deprived) | 0.207 | 0.228 | 0.217 | 0.229 | 0.221 | 0.250 |
| 2 | 0.222 | 0.231 | 0.223 | 0.228 | 0.232 | 0.245 |
| 3 | 0.230 | 0.218 | 0.225 | 0.213 | 0.225 | 0.227 |
| 4 | 0.188 | 0.176 | 0.187 | 0.184 | 0.184 | 0.167 |
| 5 (most deprived) | 0.153 | 0.147 | 0.147 | 0.146 | 0.138 | 0.111 |

**Notes.**

* Sample of patients undergone a hip replacement (excluding bilateral operations and revisions) in NHS hospitals who did not provide pre-operative responses excluding missing values on age, sex, rurality, inpatient waiting times, and symptom duration.

** Sample of patients undergone a hip replacement (excluding bilateral operations, revisions) in NHS hospitals who provided pre-operative responses, and were linked to outpatient appointments, excluding missing values on age, sex, rurality, inpatient waiting times, and symptom duration (Regression sample in Table A1).

**Table A4. Regression results: Inpatient waiting times**

|  |  | Interaction with | | | | |
| --- | --- | --- | --- | --- | --- | --- |
| Variable | Baseline | 2016 | 2017 | 2018 | 2019 | 2020 |
| Pre-operative Oxford Hip Score |  |  |  |  |  |  |
| 0-4 | -6.50*** | -1.74 | -0.07 | -4.91* | -4.79* | -11.87** |
|  | (2.00) | (2.70) | (2.69) | (2.64) | (2.79) | (4.63) |
| 5-8 | -2.72** | -0.26 | -1.30 | -2.20 | -5.92*** | -20.22*** |
|  | (1.34) | (1.83) | (1.84) | (1.82) | (1.92) | (3.19) |
| 9-12 | -2.31* | 0.48 | -0.81 | -0.23 | -0.61 | -2.06 |
|  | (1.23) | (1.68) | (1.69) | (1.68) | (1.77) | (2.97) |
| 13-16 (ref. group) |  |  |  |  |  |  |
| 17-20 | 1.35 | 0.48 | 0.71 | 0.65 | 0.50 | 3.88 |
|  | (1.21) | (1.66) | (1.69) | (1.68) | (1.77) | (3.19) |
| 21-24 | 2.97** | 0.51 | 0.35 | -0.34 | 0.12 | 0.28 |
|  | (1.31) | (1.78) | (1.83) | (1.83) | (1.91) | (3.41) |
| 25-28 | 5.42*** | -1.90 | -0.62 | -0.49 | -1.07 | 6.46 |
|  | (1.47) | (2.02) | (2.11) | (2.06) | (2.20) | (3.95) |
| 29-32 | 4.66** | 0.25 | 2.25 | 3.03 | 1.40 | 24.72*** |
|  | (1.82) | (2.50) | (2.60) | (2.59) | (2.71) | (4.96) |
| 33-48 | 8.07*** | 2.56 | 4.06 | 1.38 | 0.18 | 1.99 |
|  | (2.06) | (2.86) | (3.05) | (2.96) | (3.09) | (5.48) |
| Symptoms duration |  |  |  |  |  |  |
| Less than a year (ref. group) |  |  |  |  |  |  |
| 1-5 years | 10.52*** | 2.07 | 4.18*** | 5.04*** | 7.45*** | 64.95*** |
|  | (1.11) | (1.54) | (1.62) | (1.61) | (1.72) | (3.79) |
| 6-10 years | 13.54*** | 2.23 | 5.01** | 5.02** | 10.76*** | 80.86*** |
|  | (1.49) | (2.05) | (2.11) | (2.10) | (2.23) | (4.55) |
| >10 years | 14.82*** | 4.33* | 5.03** | 5.26** | 11.87*** | 64.99*** |
|  | (1.76) | (2.42) | (2.48) | (2.46) | (2.62) | (5.02) |
| Male (ref Female) | -0.61 | 0.06 | -0.49 | 0.22 | -0.64 | 0.59 |
|  | (0.77) | (1.06) | (1.08) | (1.06) | (1.13) | (1.98) |
| Age |  |  |  |  |  |  |
| 18-44 | 9.20*** | -3.98 | -2.26 | -5.32 | -5.82* | 1.01 |
|  | (2.37) | (3.27) | (3.32) | (3.31) | (3.51) | (5.57) |
| 45-54 | 4.53*** | -0.79 | -1.33 | -2.30 | -1.96 | -15.31*** |
|  | (1.45) | (1.97) | (2.00) | (2.00) | (2.12) | (3.57) |
| 55-64 | 2.23** | -0.47 | 0.85 | -0.55 | -1.24 | -12.36*** |
|  | (1.02) | (1.40) | (1.43) | (1.41) | (1.48) | (2.56) |
| 65-74 (ref. group) |  |  |  |  |  |  |
| 75-84 | -1.06 | 1.49 | 1.35 | 1.54 | 0.55 | 3.87 |
|  | (0.94) | (1.29) | (1.32) | (1.30) | (1.37) | (2.45) |
| >=85 | 2.86 | -1.60 | -5.23** | -4.34* | -6.43** | 8.31* |
|  | (1.80) | 2.42 | 2.48 | 2.45 | 2.59 | 4.71 |
| Ethnicity |  |  |  |  |  |  |
| White (ref. group) |  |  |  |  |  |  |
| Mixed | -5.64 | 13.18 | 10.44 | 0.45 | 23.90* | 16.37 |
|  | (7.55) | (10.52) | (11.70) | (10.87) | (12.21) | (23.19) |
| Asian | 4.28 | -3.23 | -1.16 | -0.45 | 0.38 | -7.49 |
|  | (5.26) | (7.32) | (7.12) | (7.24) | (7.55) | (11.14) |
| Black | 3.7 | -8.47 | -1.57 | 7.83 | -8.27 | 17.16 |
|  | (4.61) | (6.51) | (6.27) | (6.29) | (6.71) | (11.45) |
| Other | -0.64 | 0.58 | 4.98 | 6.30 | -0.94 | -53.80*** |
|  | (5.55) | (7.46) | (7.49) | (7.79) | (7.96) | (16.08) |
| Missing | -2.33* | 0.28 | 0.43 | 1.33 | -0.27 | 3.18 |
|  | (1.33) | (1.81) | (1.83) | (1.80) | (1.89) | (2.96) |
| Rural (ref group: urban) | 0.05 | 0.18 | 0.01 | 0.60 | -0.40 | -7.44*** |
|  | (0.94) | (1.27) | (1.29) | (1.28) | (1.34) | (2.24) |
| Emergency admissions^ |  |  |  |  |  |  |
| 0 (ref. group) |  |  |  |  |  |  |
| 1 | -1.97 | -1.65 | -1.21 | -3.10 | -1.23 | -17.34*** |
|  | (1.52) | (2.06) | (2.07) | (2.03) | (2.13) | (3.97) |
| >=2 | -3.18 | -4.55 | -2.44 | -6.77* | -3.34 | -39.12*** |
|  | (2.90) | (3.90) | (4.00) | (3.94) | (3.99) | (7.38) |
| Primary diagnosis^^ |  |  |  |  |  |  |
| Coxarthr. unsp. (ref. group) |  |  |  |  |  |  |
| Other primary coxarthrosis | 0.10 | 2.94 | 2.68 | -1.98 | 3.20 | 8.03 |
|  | (2.11) | (2.85) | (3.02) | (2.98) | (3.21) | (6.08) |
| Polyarthrosis unsp. | 1.15 | -0.36 | 0.02 | -1.28 | 0.18 | -13.47*** |
|  | (1.61) | (2.15) | (2.16) | (2.13) | (2.26) | (4.02) |
| Osteonecrosis unsp. | -7.15* | -3.62 | -11.35** | -7.99 | -5.73 | -13.37 |
|  | (3.68) | (4.93) | (5.02) | (4.97) | (5.50) | (9.08) |
| Primary cox., bilateral | 4.01 | 3.74 | -2.06 | -4.31 | 7.33 | -29.06** |
|  | (3.18) | (4.85) | (5.51) | (5.26) | (5.87) | (12.72) |
| Secondary diagnosis^^^ |  |  |  |  |  |  |
| Hypertension uncompl. | 0.83 | -0.08 | 0.33 | -0.05 | 2.83** | 2.44 |
|  | (0.79) | (1.08) | (1.11) | (1.09) | (1.15) | (2.04) |
| Obesity | 2.61** | -0.71 | 0.13 | -2.20 | -1.36 | 11.19*** |
|  | (1.09) | (1.46) | (1.45) | (1.41) | (1.46) | (2.39) |
| Chronic pulmonary dis. | 2.27** | -0.51 | 2.03 | 0.62 | 1.14 | 3.05 |
|  | (0.99) | (1.35) | (1.37) | (1.35) | (1.42) | (2.47) |
| Diabetes uncompl. | 3.66*** | -1.01 | -2.09 | 0.45 | -0.75 | -0.04 |
|  | (1.20) | (1.64) | (1.67) | (1.65) | (1.74) | (3.14) |
| Cardiac arrhythmias | 6.32*** | -2.64 | -1.66 | -1.82 | -0.59 | 10.85*** |
|  | (1.31) | (1.78) | (1.78) | (1.76) | (1.85) | (3.30) |
| Hypothyroidism | 2.12 | -0.90 | -1.61 | -0.13 | -1.01 | -3.36 |
|  | (1.35) | (1.82) | (1.85) | (1.81) | (1.87) | (3.3) |
| Depression | 3.59** | -1.16 | -1.86 | -3.38* | -1.20 | -6.04* |
|  | (1.56) | (2.10) | (2.07) | (2.03) | (2.10) | (3.33) |
| Renal failure | 2.00 | 1.37 | -0.49 | -0.11 | 1.73 | -11.94*** |
|  | (1.51) | (2.05) | (2.06) | (2.03) | (2.09) | (3.78) |
| Rheumatoid arthritis | -1.09 | -1.02 | 2.80 | 3.25 | 0.13 | 7.96** |
|  | (1.61) | (2.16) | (2.16) | (2.14) | (2.24) | (3.68) |
| Valvular disease | 5.53** | -0.26 | -1.70 | 1.90 | -2.30 | 7.41 |
|  | (2.24) | (2.99) | (2.97) | (2.92) | (3.02) | (5.67) |
| Index of Multiple Deprivation (IMD) quintile |  |  |  |  |  |  |
| 1 (ref. group: least deprived) |  |  |  |  |  |  |
| 2 | -0.88 | 1.40 | 2.33 | 1.37 | 1.91 | 5.80** |
|  | (1.09) | (1.49) | (1.52) | (1.50) | (1.59) | (2.66) |
| 3 | 0.54 | -1.14 | 1.61 | -0.30 | 0.83 | 7.48*** |
|  | (1.13) | (1.55) | (1.58) | (1.56) | (1.65) | (2.76) |
| 4 | 1.46 | -0.82 | 0.85 | 1.37 | -0.95 | -0.21 |
|  | (1.21) | (1.66) | (1.68) | (1.67) | (1.75) | (3.05) |
| 5 (most deprived) | 2.50* | -2.74 | 0.35 | 1.78 | 1.96 | 17.91*** |
|  | (1.32) | (1.82) | (1.86) | (1.84) | (1.94) | (3.59) |
| Year |  |  |  |  |  |  |
| 2015 (ref) |  |  |  |  |  |  |
| 2016 | 35.21 |  |  |  |  |  |
|  | (24.30) |  |  |  |  |  |
| 2017 | 27.93 |  |  |  |  |  |
|  | (25.99) |  |  |  |  |  |
| 2018 | 97.03*** |  |  |  |  |  |
|  | (15.03) |  |  |  |  |  |
| 2019 | 25.23 |  |  |  |  |  |
|  | (17.00) |  |  |  |  |  |
| 2020 | 91.01*** |  |  |  |  |  |
|  | (18.61) |  |  |  |  |  |
| Month |  |  |  |  |  |  |
| January (ref) |  |  |  |  |  |  |
| February | 1.28 |  |  |  |  |  |
|  | (0.79) |  |  |  |  |  |
| March | -2.87*** |  |  |  |  |  |
|  | (0.78) |  |  |  |  |  |
| April | -7.12*** |  |  |  |  |  |
|  | (0.78) |  |  |  |  |  |
| May | -6.63*** |  |  |  |  |  |
|  | (0.76) |  |  |  |  |  |
| June | -7.44*** |  |  |  |  |  |
|  | (0.76) |  |  |  |  |  |
| July | -6.80*** |  |  |  |  |  |
|  | (0.77) |  |  |  |  |  |
| August | -7.96*** |  |  |  |  |  |
|  | (0.79) |  |  |  |  |  |
| September | -8.15*** |  |  |  |  |  |
|  | (0.77) |  |  |  |  |  |
| October | -2.83*** |  |  |  |  |  |
|  | (0.78) |  |  |  |  |  |
| November | 1.12 |  |  |  |  |  |
|  | (0.77) |  |  |  |  |  |
| December | 2.65*** |  |  |  |  |  |
|  | (0.82) |  |  |  |  |  |
| Constant | 214.13*** |  |  |  |  |  |
|  | (40.47) |  |  |  |  |  |

**Notes.** Standard errors in parentheses; *p<0.1, ** p<0.05, *** p<0.01

Mean values for all listed variables (except for the two labelled “median”) over six financial years.

^ Emergency admissions in the year preceding a hip replacement surgery.

^^ Indicators for the most frequent primary diagnosis at admission and an indicator for any other primary diagnosis. We only show the most common five primary diagnoses; other diagnoses included in the models are: other secondary coxarthrosis, other dyspalstic coxarthrosis, gonarthrosis (unspecified), arthritis (unspecified), other post-traumatic coxarth., other.

^^^ Indicators for each of the Elixhauser morbidities recorded in the year preceding the hip replacement surgery. We only show the most common ten secondary diagnoses; other diagnoses included: fluid & electrolyte disorder, alcohol abuse, other neurological disorder, congenital heart failure, peripheral vascular disorder, deficiency anemia, solid tumor w/o met., liver disease, coagulopathy, diabetes complicated, peptic ulcer, weight loss, pulmonary circulation disorder, metastatic cancer, lymphoma, drug abuse, paralysis, psychoses, hypertension (complicated), blood loss anaemia.

**Table A5. Wald tests: Inpatient waiting times**

|  | 2015 vs 2016 | 2016 vs 2017 | 2017 vs 2018 | 2018 vs 2019 | 2019 vs 2020 | 2015 vs 2020 |
| --- | --- | --- | --- | --- | --- | --- |
|  | F-test^ | F-test^^ | F-test^^ | F-test^^ | F-test^^ | F-test^^^ |
|  | (P-value) | (P-value) | (P-value) | (P-value) | (P-value) | (P-value) |
| Pre-operative Oxford Hip Score | | | | | |  |
| 0-4 | 0.418 | 0.435 | 3.805* | 0.002 | 2.366 | 6.567** |
|  | (0.518) | (0.510) | (0.051) | (0.962) | (0.124) | (0.010) |
| 5-8 | 0.020 | 0.345 | 0.262 | 4.079** | 19.921*** | 40.167*** |
|  | (0.887) | (0.557) | (0.609) | (0.043) | (0.000) | (0.000) |
| 9-12 | 0.082 | 0.629 | 0.130 | 0.049 | 0.235 | 0.480 |
|  | (0.775) | (0.428) | (0.719) | (0.824) | (0.628) | (0.489) |
| 13-16 (ref group) |  |  |  |  |  |  |
| 17-20 | 0.084 | 0.020 | 0.001 | 0.008 | 1.100 | 1.479 |
|  | (0.771) | (0.888) | (0.971) | (0.928) | (0.294) | (0.224) |
| 21-24 | 0.083 | 0.009 | 0.142 | 0.057 | 0.002 | 0.007 |
|  | (0.773) | (0.924) | (0.706) | (0.811) | (0.962) | (0.934) |
| 25-28 | 0.884 | 0.390 | 0.004 | 0.071 | 3.526* | 2.678 |
|  | (0.347) | (0.532) | (0.951) | (0.790) | (0.060) | (0.102) |
| 29-32 | 0.010 | 0.631 | 0.090 | 0.361 | 21.477*** | 24.800*** |
|  | (0.921) | (0.427) | (0.764) | (0.548) | (0.000) | (0.000) |
| 33-48 | 0.802 | 0.247 | 0.747 | 0.144 | 0.105 | 0.132 |
|  | (0.370) | (0.619) | (0.387) | (0.704) | (0.746) | (0.716) |

**Notes**. P-values in parentheses; *p<0.1, ** p<0.05, *** p<0.01

^ The tested hypothesis is H_0_: OHS-category*year_2016_=0. The reported F-tests are equivalent with the square of the t-statistics derived the second column (labelled 2016) of Table A5. For instance, (1.743/2.696)^2=0.418

^^ The tested hypotheses is H_0_: OHS-category*year_t+1_=OHS-category*year_t_ for comparing consecutive years (t=2016 to 2019).

^^^ The tested hypothesis is H_0_: OHS-category*year_2020_=0. The reported F-tests are equivalent with the square of the t-statistics derived the second column (labelled 2020) of Table A5. For instance, (11.872/4.633)^2=6.567

**Table A6. Wald tests: Outpatient waiting times**

|  | 2015 vs 2016 | 2016 vs 2017 | 2017 vs 2018 | 2018 vs 2019 | 2019 vs 2020 | 2015 vs 2020 |
| --- | --- | --- | --- | --- | --- | --- |
|  | F-test | F-test | F-test | F-test | F-test | F-test |
|  | (P-value) | (P-value) | (P-value) | (P-value) | (P-value) | (P-value) |
| Pre-operative Oxford Hip Score | | | | | |  |
| 0-4 | 1.046 | 2.904* | 0.047 | 0.927 | 1.046 | 0.490 |
|  | (0.306) | (0.088) | (0.828) | (0.336) | (0.306) | (0.484) |
| 5-8 | 1.549 | 0.078 | 0.042 | 0.451 | 2.385 | 0.244 |
|  | (0.213) | (0.780) | (0.838) | (0.502) | (0.123) | (0.621) |
| 9-12 | 0.134 | 2.233 | 3.609* | 0.042 | 0.005 | 0.001 |
|  | (0.714) | (0.135) | (0.057) | (0.838) | (0.946) | (0.974) |
| 13-16 (ref group) |  |  |  |  |  |  |
| 17-20 | 0.042 | 0.444 | 1.014 | 0.191 | 0.851 | 0.593 |
|  | (0.838) | (0.505) | (0.314) | (0.662) | (0.356) | (0.441) |
| 21-24 | 1.983 | 0.506 | 2.145 | 0.606 | 0.212 | 0.193 |
|  | (0.159) | (0.477) | (0.143) | (0.436) | (0.645) | (0.661) |
| 25-28 | 2.429 | 0.233 | 1.182 | 0.223 | 0.008 | 0.019 |
|  | (0.119) | (0.630) | (0.277) | (0.636) | (0.927) | (0.890) |
| 29-32 | 0.851 | 0.004 | 0.271 | 0.054 | 4.393** | 2.201 |
|  | (0.356) | (0.951) | (0.602) | (0.817) | (0.036) | (0.138) |
| 33-48 | 0.585 | 0.017 | 0.140 | 0.097 | 1.149 | 1.023 |
|  | (0.444) | (0.897) | (0.708) | (0.755) | (0.284) | (0.312) |

**Notes**. P-values in parentheses; *p<0.1, ** p<0.05, *** p<0.01

^ The tested hypothesis is H_0_: OHS-category*year_2016_=0. The reported F-tests are equivalent with the square of the t-statistics derived the second column (labelled 2016) of Table A6.

^^ The tested hypotheses is H_0_: OHS-category*year_t+1_=OHS-category*year_t_ for comparing consecutive years (t=2016 to 2019).

^^^ The tested hypothesis is H_0_: OHS-category*year_2020_=0. The reported F-tests are equivalent with the square of the t-statistics derived the second column (labelled 2020) of Table A6.

**Table A7. Regression results: Outpatient waiting times**

|  |  | Interaction with | | | | | |
| --- | --- | --- | --- | --- | --- | --- | --- |
| Variable | Baseline | 2016 | 2017 | 2018 | 2019 | 2020 |  |
| Pre-operative Oxford Hip Score |  |  |  |  |  |  |  |
| 0-4 | -3.38 | 4.66 | -2.65 | -1.74 | 2.48 | -5.49 |  |
|  | (3.39) | (4.56) | (4.54) | (4.47) | (4.72) | (7.84) |  |
| 5-8 | -6.34*** | 3.84 | 3.01 | 3.62 | 5.71* | -2.67 |  |
|  | (2.27) | (3.09) | (3.11) | (3.07) | (3.25) | (5.40) |  |
| 9-12 | -2.78 | 1.04 | 5.16* | -0.09 | 0.51 | 0.17 |  |
|  | (2.08) | (2.84) | (2.86) | (2.84) | (2.99) | (5.02) |  |
| 13-16 (ref. group) |  |  |  |  |  |  |  |
| 17-20 | 2.60 | 0.57 | 2.43 | -0.41 | 0.88 | -4.16 |  |
|  | (2.04) | (2.80) | (2.86) | (2.84) | (3.00) | (5.4) |  |
| 21-24 | 3.76* | 4.25 | 2.13 | -2.34 | 0.15 | -2.54 |  |
|  | (2.21) | (3.02) | (3.09) | (3.09) | (3.24) | (5.78) |  |
| 25-28 | 2.13 | 5.32 | 3.65 | -0.2 | 1.54 | 0.92 |  |
|  | (2.49) | (3.41) | (3.57) | (3.49) | (3.71) | (6.68) |  |
| 29-32 | 3.17 | 3.89 | 4.15 | 6.46 | 5.39 | -12.46 |  |
|  | (3.09) | (4.22) | (4.40) | (4.39) | (4.58) | (8.40) |  |
| 33-48 | 7.22** | 3.7 | 4.36 | 2.40 | 0.74 | -9.38 |  |
|  | (3.48) | (4.84) | (5.16) | (5.01) | (5.24) | (9.27) |  |
| Symptoms duration |  |  |  |  |  |  |  |
| Less than a year (ref. group) |  |  |  |  |  |  |  |
| 1-5 years | 15.68*** | 0.75 | 0.23 | 0.82 | 1.86 | -0.22 |  |
|  | (1.89) | (2.61) | (2.74) | (2.73) | (2.92) | (6.41) |  |
| 6-10 years | 19.92*** | -1.38 | -0.48 | 2.03 | 3.34 | 1.17 |  |
|  | (2.52) | (3.46) | (3.57) | (3.55) | (3.77) | (7.7) |  |
| >10 years | 14.29*** | 3.06 | 3.32 | 7.20* | 5.36 | -0.89 |  |
|  | (2.98) | (4.09) | (4.19) | (4.17) | (4.44) | (8.49) |  |
| Male (ref Female) | -1.79 | 0.23 | -0.38 | 0.07 | 1.68 | -3.25 |  |
|  | (1.31) | (1.79) | (1.83) | (1.80) | (1.91) | (3.35) |  |
| Age |  |  |  |  |  |  |  |
| 18-44 | 15.73*** | -11.97 | -10.82* | -9.85* | -10.16* | 5.35 |  |
|  | (4.03) | (5.56) | (5.64) | (5.62) | (5.97) | (9.45) |  |
| 45-54 | 6.52*** | -3.44 | -4.66 | -6.64* | -5.01 | 3.74 |  |
|  | (2.46) | (3.34) | (3.39) | (3.40) | (3.60) | (6.07) |  |
| 55-64 | 1.05 | 3.17 | 2.97 | -1.00 | 0.59 | 3.53 |  |
|  | (1.73) | (2.37) | (2.43) | (2.39) | (2.51) | (4.33) |  |
| 65-74 (ref. group) |  |  |  |  |  |  |  |
| 75-84 | -2.75* | -1.20 | 1.24 | -0.80 | 0.56 | -4.35 |  |
|  | (1.59) | (2.18) | (2.23) | (2.20) | (2.32) | (4.14) |  |
| >=85 | -1.71 | -2.59 | -2.08 | -5.67 | 1.04 | -14.76* |  |
|  | (3.05) | (4.10) | (4.20) | (4.14) | (4.37) | (7.98) |  |
| Ethnicity |  |  |  |  |  |  |  |
| White (ref. group) |  |  |  |  |  |  |  |
| Mixed | 10.69 | -19.83 | -28.86 | -8.38 | -6.48 | -30.75 |  |
|  | (12.77) | (17.80) | (19.80) | (18.39) | (20.65) | (39.26) |  |
| Asian | -0.03 | -10.21 | 16.30 | 5.12 | -2.27 | -15.67 |  |
|  | (8.90) | (12.38) | (12.04) | (12.24) | (12.77) | (18.84) |  |
| Black | 12.50 | -12.58 | -5.28 | 0.96 | -4.18 | -26.20 |  |
|  | (7.80) | (11.02) | (10.60) | (10.63) | (11.35) | (19.38) |  |
| Other | -6.10 | 5.11 | -4.63 | 21.91* | 2.62 | -3.66 |  |
|  | (9.38) | (12.62) | (12.67) | (13.17) | (13.47) | (27.21) |  |
| Missing | -4.63** | -1.59 | -1.49 | 2.56 | -0.70 | 0.01 |  |
|  | (2.25) | (3.07) | (3.11) | (3.05) | (3.21) | (5.01) |  |
| Rural (ref group: urban) | -1.00 | 0.78 | 0.08 | 1.43 | -1.71 | 3.99 |  |
|  | (1.59) | (2.15) | (2.19) | (2.16) | (2.27) | (3.79) |  |
| Emergency admissions^ |  |  |  |  |  |  |  |
| 0 (ref. group) |  |  |  |  |  |  |  |
| 1 | -4.72* | 0.19 | -6.02* | -4.96 | -3.01 | 7.84 |  |
|  | (2.57) | (3.48) | (3.51) | (3.44) | (3.60) | (6.72) |  |
| >=2 | -17.25*** | 8.85 | 9.81 | 2.21 | 4.91 | 9.41 |  |
|  | (4.91) | (6.60) | (6.77) | (6.68) | (6.76) | (12.5) |  |
| Primary diagnosis^^ |  |  |  |  |  |  |  |
| Cox. unsp. (ref. group) |  |  |  |  |  |  |  |
| Other primary cox. | 3.78 | -2.70 | -2.61 | -7.59 | -0.67 | 1.61 |  |
|  | (3.58) | (4.82) | (5.11) | (5.05) | (5.42) | (10.28) |  |
| Polyarthrosis unsp. | 0.95 | -1.66 | -1.62 | 1.59 | -3.85 | -8.43 |  |
|  | (2.72) | (3.64) | (3.65) | (3.61) | (3.82) | (6.79) |  |
| Osteonecrosis unsp. | -10.33* | -5.41 | -0.19 | -2.18 | -26.89*** | -24.22 |  |
|  | (6.23) | (8.34) | (8.49) | (8.41) | (9.31) | (15.35) |  |
| Primary cox., bilateral | 5.51 | -11.32 | -10.59 | -15.79* | -8.70 | -20.62 |  |
|  | (5.37) | (8.21) | (9.31) | (8.90) | (9.92) | (21.54) |  |
| Secondary diagnosis^^^ |  |  |  |  |  |  |  |
| Hypertension uncompl. | 0.56 | 0.26 | -0.08 | 1.36 | -1.23 | 8.70** |  |
|  | (1.33) | (1.83) | (1.87) | (1.84) | (1.95) | (3.45) |  |
| Obesity | 3.14* | -2.84 | -2.13 | -1.29 | 2.22 | -5.98 |  |
|  | (1.85) | (2.47) | (2.46) | (2.39) | (2.46) | (4.05) |  |
| Chronic pulmonary dis. | 3.31** | -2.66 | -0.86 | -2.01 | -6.74*** | -1.90 |  |
|  | (1.68) | (2.28) | (2.32) | (2.28) | (2.40) | (4.18) |  |
| Diabetes uncompl. | 0.69 | 0.18 | 1.97 | -0.88 | 4.47 | 2.63 |  |
|  | (2.02) | (2.77) | (2.82) | (2.79) | (2.94) | (5.31) |  |
| Cardiac arrhythmias | 2.66 | 0.19 | -1.11 | -1.11 | -1.12 | -0.37 |  |
|  | (2.22) | (3.01) | (3.02) | (2.98) | (3.13) | (5.59) |  |
| Hypothyroidism | 1.64 | -2.28 | -2.58 | -3.12 | -3.24 | 6.39 |  |
|  | (2.29) | (3.08) | (3.13) | (3.07) | (3.17) | (5.58) |  |
| Depression | -3.37 | -1.15 | 3.51 | 3.67 | 5.12 | -7.71 |  |
|  | (2.64) | (3.55) | (3.49) | (3.43) | (3.55) | (5.64) |  |
| Renal failure | -1.28 | 1.29 | -2.51 | 4.23 | 3.57 | -2.39 |  |
|  | (2.55) | (3.47) | (3.49) | (3.43) | (3.54) | (6.39) |  |
| Rheumatoid arthritis | -0.16 | -3.02 | -0.50 | 0.56 | -0.66 | 3.24 |  |
|  | (2.72) | (3.66) | (3.66) | (3.63) | (3.8) | (6.25) |  |
| Valvular disease | -3.97 | 7.79 | 2.06 | 4.65 | 9.16* | -0.42 |  |
|  | (3.80) | (5.06) | (5.03) | (4.94) | (5.10) | (9.60) |  |
| Index of Multiple Deprivation (IMD) quintile |  |  |  |  |  |  |  |
| 1 (ref. group: least deprived) |  |  |  |  |  |  |  |
| 2 | -1.67 | 2.03 | 3.94 | 0.08 | 5.72** | 3.38 |  |
|  | (1.85) | (2.53) | (2.58) | (2.54) | (2.68) | (4.50) |  |
| 3 | 0.49 | 1.99 | 2.07 | -4.23 | 1.30 | 0.91 |  |
|  | (1.92) | (2.62) | (2.67) | (2.63) | (2.79) | (4.66) |  |
| 4 | -1.78 | 4.82* | 5.90** | 0.62 | 3.36 | 1.42 |  |
|  | (2.04) | (2.80) | (2.85) | (2.83) | (2.97) | (5.16) |  |
| 5 (most deprived) | -0.56 | 4.61 | 0.02 | 2.10 | 5.44* | 2.99 |  |
|  | (2.24) | (3.08) | (3.15) | (3.11) | (3.28) | (6.07) |  |
| Referral source |  |  |  |  |  |  |  |
| GP referral (ref. group) |  |  |  |  |  |  |  |
| Responsible consultant | 19.75*** | -6.85 | -14.60*** | 0.48 | 12.07** | -18.02* |  |
|  | (3.92) | (5.41) | (5.55) | (5.48) | (5.97) | (10.69) |  |
| Other | 8.25*** | -4.40* | -4.61* | -4.66** | -1.15 | 7.56* |  |
|  | (1.77) | (2.39) | (2.40) | (2.36) | (2.48) | (4.17) |  |
| N. of outpatient appointments |  |  |  |  |  |  |  |
| 1 (ref. group) |  |  |  |  |  |  |  |
| 2 | 86.50*** | 1.08 | 1.04 | 2.09 | -3.44 | -2.20 |  |
|  | (1.76) | (2.41) | (2.46) | (2.41) | (2.55) | (4.28) |  |
| 3 | 191.30*** | -4.83 | -1.00 | 1.70 | -8.59** | -18.32*** |  |
|  | (2.40) | (3.27) | (3.34) | (3.27) | (3.48) | (5.75) |  |
| >3 | 354.37*** | -14.82*** | 3.99 | -0.48 | -12.33*** | -15.59*** |  |
|  | (2.07) | (2.83) | (2.86) | (2.84) | (2.97) | (4.92) |  |
| Year |  |  |  |  |  |  |  |
| 2015 (ref) |  |  |  |  |  |  |  |
| 2016 | 14.87 |  |  |  |  |  |  |
|  | (41.12) |  |  |  |  |  |  |
| 2017 | -3.15 |  |  |  |  |  |  |
|  | (43.98) |  |  |  |  |  |  |
| 2018 | -18.31 |  |  |  |  |  |  |
|  | (25.43) |  |  |  |  |  |  |
| 2019 | -17.78 |  |  |  |  |  |  |
|  | (28.76) |  |  |  |  |  |  |
| 2020 | -26.76 |  |  |  |  |  |  |
|  | (31.53) |  |  |  |  |  |  |
| Month |  |  |  |  |  |  |  |
| January (ref) |  |  |  |  |  |  |  |
| February | -3.83*** |  |  |  |  |  |  |
|  | (1.34) |  |  |  |  |  |  |
| March | -3.02** |  |  |  |  |  |  |
|  | (1.32) |  |  |  |  |  |  |
| April | -0.60 |  |  |  |  |  |  |
|  | (1.31) |  |  |  |  |  |  |
| May | 1.11 |  |  |  |  |  |  |
|  | (1.29) |  |  |  |  |  |  |
| June | -0.23 |  |  |  |  |  |  |
|  | (1.28) |  |  |  |  |  |  |
| July | 2.04 |  |  |  |  |  |  |
|  | (1.30) |  |  |  |  |  |  |
| August | 3.23** |  |  |  |  |  |  |
|  | (1.33) |  |  |  |  |  |  |
| September | 5.05*** |  |  |  |  |  |  |
|  | (1.31) |  |  |  |  |  |  |
| October | 2.97** |  |  |  |  |  |  |
|  | (1.31) |  |  |  |  |  |  |
| November | 0.18 |  |  |  |  |  |  |
|  | (1.31) |  |  |  |  |  |  |
| December | -1.51 |  |  |  |  |  |  |
|  | (1.39) |  |  |  |  |  |  |
| Constant | 135.02** |  |  |  |  |  |  |
|  | (68.49) |  |  |  |  |  |  |

**Notes.** Standard errors in parentheses; *p<0.1, ** p<0.05, *** p<0.01

Mean values for all listed variables (except for the two labelled “median”) over six financial years.

^ Emergency admissions in the year preceding a hip replacement surgery.

^^ Indicators for the most frequent primary diagnosis at admission and an indicator for any other primary diagnosis. We only show the most common five primary diagnoses; other diagnoses included in the models are: other secondary coxarthrosis, other dyspalstic coxarthrosis, gonarthrosis (unspecified), arthritis (unspecified), other post-traumatic coxarth., other.

^^^ Indicators for each of the Elixhauser morbidities recorded in the year preceding the hip replacement surgery. We only show the most common ten secondary diagnoses; other diagnoses included: fluid & electrolyte disorder, alcohol abuse, other neurological disorder, congenital heart failure, peripheral vascular disorder, deficiency anemia, solid tumor w/o met., liver disease, coagulopathy, diabetes complicated, peptic ulcer, weight loss, pulmonary circulation disorder, metastatic cancer, lymphoma, drug abuse, paralysis, psychoses, hypertension (complicated), blood loss anaemia.

**Table A8. Marginal effects for outpatient waiting times (base model and alternative models)**

|  | 2015/16 | 2016/17 | 2017/18 | 2018/19 | | 2019/20 | | 2020/21 | |  |
| --- | --- | --- | --- | --- | --- | --- | --- | --- | --- | --- |
| N. of Obs. | (21,192) | (24,059) | (22,166) | (23,233) | | (18,956) | | (4,414) | |  |
| **Base model^** |  |  |  |  | |  | |  | |  |
| *Pre-operative Oxford Hip Score* | |  |  |  | |  | |  | |  |
| 0-4 | -3.38 | 1.29 | -6.03** | -5.12* | | -0.89 | | -8.87 | |  |
| 5-8 | -6.34*** | -2.49 | -3.33 | -2.72 | | -0.63 | | -9.01* | |  |
| 9-12 | -2.78 | -1.74 | 2.38 | -2.87 | | -2.28 | | -2.62 | |  |
| 13-16 (ref. group) |  |  |  |  | |  | |  | |  |
| 17-20 | 2.60 | 3.18* | 5.03** | 2.20 | | 3.48 | | -1.56 | |  |
| 21-24 | 3.76* | 8.01*** | 5.89*** | 1.42 | | 3.91* | | 1.22 | |  |
| 25-28 | 2.13 | 7.45*** | 5.78** | 1.93 | | 3.67 | | 3.05 | |  |
| 29-32 | 3.17 | 7.06** | 7.32** | 9.63*** | | 8.56** | | -9.29 | |  |
| 33-48 | 7.22** | 10.92*** | 11.58*** | 9.62*** | | 7.96** | | -2.16 | |  |
| N. of Obs. | (21,192) | (24,059) | (22,166) | | (23,233) | | (18,956) | | (4,414) | |
| **Partial adjustment^^** |  | |  | |  | |  | |  | |
| 0-4 | -2.74 | 0.62 | -5.04 | | -2.34 | | 0.11 | | -9.29 | |
| 5-8 | -6.77* | -2.92 | 0.69 | | -1.87 | | -0.86 | | -11.84 | |
| 9-12 | -6.03* | 1.21 | 1.79 | | -2.10 | | -2.18 | | -0.68 | |
| 13-16 (ref. group) |  |  |  | |  | |  | |  | |
| 17-20 | -1.29 | 2.81 | 7.12** | | 3.62 | | 9.48*** | | 1.02 | |
| 21-24 | 3.49 | 11.97*** | 9.53*** | | 2.41 | | 6.03 | | 4.28 | |
| 25-28 | 6.94* | 9.16** | 9.84** | | 10.89*** | | 6.64 | | 4.41 | |
| 29-32 | 8.32* | 15.02*** | 15.23*** | | 19.60*** | | 19.29*** | | 2.28 | |
| 33-48 | 20.33*** | 25.79*** | 33.83*** | | 25.80*** | | 33.28*** | | 0.31 | |
| N. of Obs. | (11,171) | (12,531) | (11,097) | (11,265) | | (9,204) | | (1,891) | |  |
| **Restricted sample^^^** | |  |  |  | |  | |  | |  |
| 0-4 | -0.28 | -3.93*** | -2.70* | -0.44 | | -1.51 | | -0.79 | |  |
| 5-8 | 1.71* | -0.91 | -0.28 | 2.30** | | -1.21 | | -5.97** | |  |
| 9-12 | -0.09 | -0.24 | 0.07 | 0.64 | | -0.82 | | -0.81 | |  |
| 13-16 (ref. group) | 0.00 | 0.00 | 0.00 | 0.00 | | 0.00 | | 0.00 | |  |
| 17-20 | 1.27 | 0.13 | 0.06 | 1.77** | | 0.16 | | -6.77*** | |  |
| 21-24 | 0.19 | 1.32 | 1.13 | 0.93 | | -0.65 | | 0.29 | |  |
| 25-28 | 0.37 | 1.09 | 0.60 | 0.37 | | 0.17 | | -3.98 | |  |
| 29-32 | -0.17 | 2.16* | 2.22* | 0.64 | | -0.85 | | -6.79* | |  |
| 33-48 | 1.31 | 2.27 | 2.00 | 0.69 | | -1.29 | | -1.75 | |  |

**Notes.** *p<0.1, ** p<0.05, *** p<0.01

Marginal effects are presented for the Oxford Hip Score categories only. The model from which these marginal effects are derived includes patient characteristics listed in Table 1, consultant fixed effects, and year and month dummy variables. Patient characteristics and consultant fixed effects are interacted with year dummy variables.

^ Patients with any type of referral and any number of outpatient appointments; model controls for source of referral and number of outpatient appointments.

^^ Same as ^ above, but the model does not control for type of referral and number of outpatient appointments.

^^^ Sample includes only referrals from a GP and with a single outpatient appointment.

**Table A9. Average inpatient waiting time (days) for public and private providers**

|  | 2015/16 | | 2016/17 | | 2017/18 | | 2018/19 | | 2019/20 | | 2020/21 | |
| --- | --- | --- | --- | --- | --- | --- | --- | --- | --- | --- | --- | --- |
|  | N | mean wait time | N | mean wait time | N | mean wait time | N | mean wait time | N | mean wait time | N | mean wait time |
| *Unbalanced sample* | | |  |  |  |  |  |  |  |  |  |  |
| Public providers | 129 | 92.8 | 131 | 106.5 | 128 | 115.1 | 127 | 125 | 122 | 127.6 | 104 | 229.2 |
| Private providers | 105 | 57.7 | 135 | 60.3 | 139 | 60.3 | 148 | 65 | 144 | 70.6 | 115 | 146.1 |
| *Balanced sample* | |  |  |  |  |  |  |  |  |  |  |  |
| Public providers | 49 | 76 | 56 | 82.4 | 47 | 85.6 | 47 | 93.1 | 49 | 95.4 | 56 | 204.8 |
| Private providers | 49 | 76.1 | 56 | 81.3 | 47 | 84.5 | 47 | 92.9 | 49 | 95.1 | 56 | 204.7 |

**Notes**: N denotes the number of providers.

**Table A10. Outpatient waiting time. Matched sample (k:m)**

*Private providers (Independent Sector)*

|  | 2015/16 | 2016/17 | 2017/18 | 2018/19 | 2019/20 | 2020/21 |
| --- | --- | --- | --- | --- | --- | --- |
| N. of observations | (6262) | (7446) | (7020) | (9555) | (8453) | (1651) |
| Mean wait time (days) | 53.9 | 58.1 | 69.4 | 60.4 | 56.6 | 96.5 |
| *Pre-operative Oxford Hip Score* | |  |  |  |  |  |
| 0-4 | -7.01 | -4.77 | -4.67 | -8.26** | 1.88 | 8.65 |
|  | (5.02) | (4.41) | (4.43) | (3.68) | (4.20) | (9.71) |
| 5-8 | -4.24* | -3.12 | -1.74 | -0.83 | -3.48* | -23.27*** |
|  | (2.34) | (2.13) | (2.15) | (1.80) | (1.97) | (4.64) |
| 9-12 | -0.17 | -1.45 | -1.30 | -0.09 | -1.32 | -7.93** |
|  | (1.95) | (1.76) | (1.76) | (1.51) | (1.61) | (3.82) |
| 13-16 (reference group) | |  |  |  |  |  |
| 17-20 | 0.93 | 0.48 | 2.46 | 2.31* | 2.32 | -0.08 |
|  | (1.79) | (1.63) | (1.64) | (1.41) | (1.51) | (3.89) |
| 21-24 | 1.72 | 0.21 | 2.05 | 3.18** | 1.60 | 1.09 |
|  | (1.88) | (1.71) | (1.76) | (1.46) | (1.60) | (4.15) |
| 25-28 | 3.15 | 3.02 | 2.15 | 5.46*** | 2.63 | 9.12* |
|  | (2.03) | (1.91) | (1.97) | (1.68) | (1.76) | (4.87) |
| 29-32 | 3.21 | 3.45 | 10.69*** | 5.57*** | 5.66*** | 3.55 |
|  | (2.43) | (2.30) | (2.50) | (2.06) | (2.17) | (6.36) |
| 33-48 | 3.05 | 8.52*** | -1.00 | 8.07*** | 3.85 | 16.40** |
|  | (2.74) | (2.61) | (2.93) | (2.49) | (2.53) | (6.99) |

*Public providers (NHS Trusts)*

|  | 2015/16 | 2016/17 | 2017/18 | 2018/19 | 2019/20 | 2020/21 |
| --- | --- | --- | --- | --- | --- | --- |
| N. of observations | (12995) | (14832) | (13654) | (15086) | (12057) | (2014) |
| Mean wait time (days) | 104.0 | 105.3 | 111.9 | 107.8 | 104.5 | 129.0 |
| *Pre-operative Oxford Hip Score* | |  |  |  |  |  |
| 0-4 | 1.78 | -2.60 | -1.99 | -5.32 | -2.32 | -7.66 |
|  | (7.09) | (6.39) | (6.47) | (5.89) | (7.26) | (18.41) |
| 5-8 | -5.38 | -1.62 | -3.12 | -4.63 | -1.01 | -14.75* |
|  | (3.27) | (3.05) | (3.13) | (2.89) | (3.33) | (8.70) |
| 9-12 | -2.14 | -3.84 | 7.09*** | -1.61 | -2.19 | -8.37 |
|  | (2.75) | (2.52) | (2.55) | (2.43) | (2.74) | (7.28) |
| 13-16 (reference group) | |  |  |  |  |  |
| 17-20 | 0.09 | 4.23* | 5.39** | 2.20 | -0.78 | -5.29 |
|  | (2.53) | (2.33) | (2.38) | (2.27) | (2.58) | (7.32) |
| 21-24 | 1.96 | 7.36*** | 7.78*** | 3.77 | 5.33* | -9.93 |
|  | (2.67) | (2.46) | (2.55) | (2.37) | (2.72) | (7.79) |
| 25-28 | 3.03 | 7.79*** | 4.74* | 3.91 | 3.58 | -1.52 |
|  | (2.88) | (2.75) | (2.88) | (2.74) | (3.01) | (9.50) |
| 29-32 | 6.69* | 1.31 | 7.06* | 15.13*** | 3.14 | -30.25** |
|  | (3.48) | (3.29) | (3.62) | (3.36) | (3.73) | (12.27) |
| 33-48 | 1.69 | 8.95** | 2.92 | 17.41*** | 16.52*** | 0.33 |
|  | (3.94) | (3.82) | (4.32) | (4.05) | (4.45) | (13.59) |

**Notes.** Standard errors in parentheses; *p<0.1, ** p<0.05, *** p<0.01

Coarsened exact k:m matching at patient level (k patients treated by private providers matched to m patients treated by public providers) on the following variables: Oxford Hip Score categories, sex, age categories, Index of Multiple Deprivation quintiles, the most common thre primary diagnosis at admission (coxarthrosis unspecified, other primary coxarthrosis, polyarthrosis unspecified), the most common six secondary diagnoses of the Elixhauser morbidities recorded in the year preceding a hip replacement surgery (hypertension uncomplicated, obesity, chronic pulmonary disease, diabetes uncomplicated, cardiac arrhythmias, hypothyroidism).

Marginal effects are presented for the Oxford Hip Score categories only. The model from which these marginal effects are derived is estimated using all patients from the matched samples and includes patient characteristics listed in Table 1, consultant fixed effects, and year and month dummy variables. Patient characteristics and consultant fixed effects are interacted with year dummy variables.

**Table A11. Inpatient waiting times. Individual hip score domains**

|  | 2015/16 | 2016/17 | 2017/18 | 2018/19 | 2019/20 | 2020/21 |
| --- | --- | --- | --- | --- | --- | --- |
| N. observations | (21,192) | (24,059) | (22,166) | (23,233) | (18,956) | (4,414) |
| Pain | -3.61*** | -3.36*** | -3.83*** | -2.47*** | -4.06*** | -8.06*** |
|  | (0.89) | (0.85) | (0.89) | (0.87) | (0.96) | (2.19) |
| Sudden pain | -0.67 | -1.01 | 0.12 | -2.43*** | -1.97** | -8.14*** |
|  | (0.92) | (0.85) | (0.88) | (0.86) | (0.96) | (2.07) |
| Night pain | -2.51*** | -1.98** | -3.20*** | -1.74** | -1.18 | -5.34*** |
|  | (0.85) | (0.79) | (0.83) | (0.81) | (0.91) | (2.00) |
| Washing | -0.39 | -0.72 | 0.45 | -0.12 | -0.78 | -6.12*** |
|  | (1.03) | (0.96) | (0.98) | (0.96) | (1.06) | (2.25) |
| Transport | -1.12 | -0.47 | -1.09 | -0.74 | -0.14 | 2.22 |
|  | (0.97) | (0.90) | (0.94) | (0.92) | (1.02) | (2.25) |
| Dressing | -0.32 | 0.20 | -0.43 | -0.08 | 0.84 | -1.32 |
|  | (0.87) | (0.81) | (0.86) | (0.84) | (0.94) | (2.08) |
| Shopping | 0.78 | -0.86 | -0.40 | 0.08 | -2.38** | -4.42** |
|  | (0.95) | (0.90) | (0.94) | (0.92) | (1.02) | (2.25) |
| Walking | -0.80 | -0.86 | -0.01 | -1.22 | -2.21** | -1.99 |
|  | (0.92) | (0.86) | (0.89) | (0.87) | (0.97) | (2.10) |
| Limping | -0.77 | -0.98 | -1.02 | -2.75*** | -3.49*** | -11.76*** |
|  | (0.85) | (0.80) | (0.84) | (0.82) | (0.91) | (2.07) |
| Stairs | 0.15 | 1.37 | -1.32 | 0.10 | -0.54 | 7.33*** |
|  | (0.98) | (0.93) | (0.96) | (0.95) | (1.05) | (2.28) |
| Standing | -0.13 | -0.13 | -0.13 | -0.13 | -0.13 | -0.13 |
|  | (0.40) | (0.40) | (0.40) | (0.40) | (0.40) | (0.40) |
| Work | -1.68* | -1.79* | -1.34 | -2.56*** | -0.19 | 0.69 |
|  | (0.98) | (0.93) | (0.98) | (0.97) | (1.07) | (2.41) |

**Notes.** *p<0.1, ** p<0.05, *** p<0.01. Hospital fixed effects are included but not presented.

Marginal effects are presented for the 12 individual domains that comprise the Oxford Hip Score. The model from which these marginal effects are derived includes patient characteristics listed in Table 1, consultant fixed effects, and year and month dummy variables. Patient characteristics and consultant fixed effects are interacted with year dummy variables.

**Table A12. Inverse Probability Weighting Marginal effects for Oxford Hip Score: inpatient waiting times**

|  | 2015/16 | 2016/17 | 2017/18 | 2018/19 | 2019/20 | 2020/21 |
| --- | --- | --- | --- | --- | --- | --- |
| N. of observations | (21,192) | (24,059) | (22,166) | (23,233) | (18,956) | (4,414) |
| *Pre-operative Oxford Hip Score* | |  |  |  |  |  |
| 0-4 | -6.54*** | -8.26*** | -6.57*** | -11.38*** | -11.40*** | -17.66** |
|  | (1.85) | (1.73) | (1.87) | (2.03) | (2.27) | (8.65) |
| 5-8 | -2.74** | -2.99*** | -4.06*** | -4.93*** | -8.66*** | -22.60*** |
|  | (1.11) | (1.12) | (1.24) | (1.34) | (1.45) | (5.85) |
| 9-12 | -2.36** | -1.82* | -3.15*** | -2.57** | -2.97** | -4.69 |
|  | (1.01) | (1.01) | (1.10) | (1.20) | (1.32) | (5.36) |
| 13-16 (reference group) | |  |  |  |  |  |
| 17-20 | 1.33 | 1.83* | 2.06* | 1.97 | 1.88 | 4.89 |
|  | (0.96) | (0.98) | (1.09) | (1.21) | (1.32) | (5.65) |
| 21-24 | 2.94*** | 3.48*** | 3.25*** | 2.58** | 3.03** | 3.40 |
|  | (1.04) | (1.04) | (1.16) | (1.30) | (1.41) | (6.06) |
| 25-28 | 5.39*** | 3.48*** | 4.84*** | 4.89*** | 4.39*** | 11.86* |
|  | (1.17) | (1.18) | (1.37) | (1.47) | (1.64) | (6.93) |
| 29-32 | 4.64*** | 4.91*** | 6.96*** | 7.69*** | 6.12*** | 28.35*** |
|  | (1.46) | (1.46) | (1.63) | (1.93) | (1.91) | (7.57) |
| 33-48 | 8.15*** | 10.59*** | 12.07*** | 9.41*** | 8.13*** | 9.96 |
|  | (1.64) | (1.69) | (2.09) | (2.03) | (2.25) | (9.37) |

**Notes.** Standard errors in parentheses; *p<0.1, ** p<0.05, *** p<0.01

Marginal effects are presented for the Oxford Hip Score categories only. The model from which these marginal effects are derived includes patient characteristics listed in Table 1, consultant fixed effects, and year and month dummy variables. Patient characteristics and consultant fixed effects are interacted with year dummy variables.

**Table A13. Poisson model marginal effects for Oxford Hip Score: inpatient waiting times**

|  | 2015/16 | 2016/17 | 2017/18 | 2018/19 | 2019/20 | 2020/21 |
| --- | --- | --- | --- | --- | --- | --- |
| N. of observations | (21,192) | (24,059) | (22,166) | (23,233) | (18,956) | (4,414) |
| *Pre-operative Oxford Hip Score* | |  |  |  |  |  |
| 0-4 | -6.29*** | -7.75*** | -6.43*** | -10.79*** | -10.88*** | -16.68* |
|  | (1.71) | (1.91) | (1.99) | (2.07) | (2.14) | (9.53) |
| 5-8 | -2.75*** | -3.01*** | -3.97*** | -4.72*** | -8.30*** | -21.06*** |
|  | (0.94) | (1.14) | (1.14) | (1.38) | (1.38) | (5.17) |
| 9-12 | -2.50** | -1.79 | -3.21*** | -2.55** | -2.95** | -4.89 |
|  | (1.13) | (1.12) | (1.18) | (1.16) | (1.35) | (5.61) |
| 13-16 (reference group) | |  |  |  |  |  |
| 17-20 | 1.34 | 1.90* | 2.04* | 2.07** | 1.97 | 4.82 |
|  | (0.86) | (1.08) | (1.08) | (1.02) | (1.41) | (6.09) |
| 21-24 | 3.00*** | 3.67*** | 3.36*** | 2.77** | 3.19** | 3.20 |
|  | (1.09) | (1.04) | (1.23) | (1.09) | (1.47) | (4.73) |
| 25-28 | 5.70*** | 3.72*** | 4.97*** | 5.29*** | 4.56*** | 10.39 |
|  | (1.13) | (1.13) | (1.29) | (1.53) | (1.72) | (7.31) |
| 29-32 | 4.72*** | 5.32*** | 7.16*** | 8.37*** | 6.47*** | 28.09*** |
|  | (1.33) | (1.49) | (1.76) | (2.02) | (1.86) | (8.00) |
| 33-48 | 8.48*** | 11.48*** | 12.55*** | 9.89*** | 8.76*** | 9.69 |
|  | (1.67) | (1.80) | (2.15) | (2.19) | (2.34) | (8.79) |

**Notes.** Standard errors in parentheses; *p<0.1, ** p<0.05, *** p<0.01

Marginal effects are presented for the Oxford Hip Score categories only. The model from which these marginal effects are derived includes patient characteristics listed in Table 1, consultant fixed effects, and year and month dummy variables. Patient characteristics and consultant fixed effects are interacted with year dummy variables. Full estimates for all covariates are provided in Appendix Table A5.

**Appendix B.**

**Hip replacement identification (See PROMS guide vol 12, Annex 1)**

Hip replacement codes include:

1. total hip replacement (any of W37, W38 or W39)
2. total prosthetic replacement of head of femur (any of W46, W47 or W48)
3. hybrid prosthetic hip replacement (any of W93, W94 or W95),
4. other hip replacement (any of W52, W53 or W54 combined with any of Z76.1, Z75.6, or Z84.3)
5. Resurfacing arthroplasty of joint (any of W581, W582 combined with any of Z76.1, Z75.6, or Z84.3)

Exclude

- Revisions (if the codes for the 3-digit codes from the above 1-4 are followed by 0, 2, 3, or 4)
- Bilateral operation codes [Z941 or (Z942 & Z943)]

**Appendix C. Oxford hip score questionnaire**

1. During the past 4 weeks, how would you describe the pain you usually had from your hip?

0 = Severe,

1 = Moderate

2 = Mild

3 = Very Mild

4 = None

Binary variable for sensitivity analysis: **Pain** is severe.

2. During the past 4 weeks, have you had any sudden, severe pain - 'shooting', 'stabbing' or 'spasms' - from the affected hip?

0 = Every day

1 = Most days

2 = Some days

3 = Only 1 or 2 days

4 = No days

Binary variable for sensitivity analysis: **Sudden pain** every day.

3. During the past 4 weeks, have you been troubled by pain from your hip in bed at night?

0 = Every night

1 = Most nights

2 = Some nights

3 = Only 1 or 2 nights

4 = No nights

Binary variable for sensitivity analysis: **Night pain** every night.

4. During the past 4 weeks, have you had trouble washing and drying yourself (all over) because of your hip?

0 = Impossible to do

1 = Extreme difficulty

2 = Moderate trouble

3 = Very little trouble

4 = No trouble at all

Binary variable for sensitivity analysis: **Washing** impossible to do or with extreme difficulty.

5. During the past 4 weeks, have you had any trouble getting in or out of your car or using public transport because of your hip?

0 = Impossible to do

1 = Extreme difficulty

2 = Moderate trouble

3 = Very little trouble

4 = No trouble at all

Binary variable for sensitivity analysis: **Transport** impossible to do or with extreme difficulty

6. During the past 4 weeks, have you been able to put on a pair of socks, stockings or tights?

0 = No, impossible

1 = With extreme difficulty

2 = With moderate difficulty

3 = With little difficulty

4 = Yes, easily

Binary variable for sensitivity analysis: **Dressing** impossible to do or with extreme difficulty.

7. During the past 4 weeks, could you do the household shopping on your own?

0 = No, impossible

1 = With extreme difficulty

2 = With moderate difficulty

3 = With little difficulty

4 = Yes, easily

Binary variable for sensitivity analysis: **Shopping** impossible to do or with extreme difficulty.

8. During the past 4 weeks, for how long have you been able to walk before pain from your hip becomes severe? (with or without a stick)

0 = Not at all pain severe on walking

1 = Around the house only

2 = 5 15 minutes

3 = 16 30 minutes

4 = No pain/more than 30 minutes

Binary variable for sensitivity analysis: **Walking** not at all or around the house only.

9. During the past 4 weeks, have you been limping when walking, because of your hip?

0 = All of the time

1 = Most of the time

2 = Often, not just at first

3 = Sometimes or just at first

4 = Rarely/Never

Binary variable for sensitivity analysis: **Limping** all of the time.

10. During the past 4 weeks, have you been able to climb a flight of stairs?

0 = No, impossible

1 = With extreme difficulty

2 = With moderate difficulty

3 = With little difficulty

4 = Yes, easily

Binary variable for sensitivity analysis: **Stairs** impossible to climb or climb with extreme difficulty.

11. During the past 4 weeks, after a meal (sat at a table), how painful has it been for you to stand up from a chair because of your hip?

0 = Unbearable

1 = Very painful

2 = Moderately painful

3 = Slightly painful

4 = not at all painful

Binary variable for sensitivity analysis: **Standing** unbearable or very painful.

12. During the past 4 weeks, how much has pain from your hip interfered with your usual work (including housework)?

0 = Totally

1 = Greatly

2 = Moderately

3 = A little bit

4 = Not at all

Binary variable for sensitivity analysis: **Work** affected by pain totally or greatly.

**Proportion of patients in each Oxford hip score domain (1 =100%)**

| OHS domain | 2015-16 | 2016-17 | 2017-18 | 2018-19 | 2019-20 | 2020-21 |
| --- | --- | --- | --- | --- | --- | --- |
| Pain | 0.57 | 0.58 | 0.61 | 0.61 | 0.61 | 0.62 |
| Sudden pain | 0.31 | 0.32 | 0.33 | 0.34 | 0.32 | 0.32 |
| Night pain | 0.45 | 0.47 | 0.50 | 0.50 | 0.49 | 0.51 |
| Washing | 0.23 | 0.24 | 0.26 | 0.27 | 0.27 | 0.29 |
| Transport | 0.36 | 0.38 | 0.40 | 0.40 | 0.40 | 0.41 |
| Dressing | 0.55 | 0.56 | 0.58 | 0.59 | 0.58 | 0.61 |
| Shopping | 0.43 | 0.44 | 0.46 | 0.46 | 0.45 | 0.51 |
| Walking | 0.36 | 0.37 | 0.38 | 0.39 | 0.38 | 0.44 |
| Limping | 0.59 | 0.59 | 0.61 | 0.62 | 0.61 | 0.66 |
| Stairs | 0.40 | 0.41 | 0.44 | 0.44 | 0.43 | 0.47 |
| Standing | 0.51 | 0.51 | 0.54 | 0.54 | 0.53 | 0.57 |
| Work | 0.66 | 0.67 | 0.70 | 0.70 | 0.69 | 0.71 |

**Appendix D. Summary statistics for private providers**

| Variable | 2015-16 | 2016-17 | 2017-18 | 2018-19 | 2019-20 | 2020-21 |
| --- | --- | --- | --- | --- | --- | --- |
| N | (7,202) | (8,598) | (8,368) | (11,299) | (10,258) | (2,648) |
| Inpatient waiting time (mean) | 52.655 | 51.066 | 52.279 | 59.064 | 66.734 | 129.379 |
| Inpatient waiting time (median) | 42 | 37 | 32 | 49 | 59 | 65 |
| Outpatient waiting time (mean) | 54.250 | 58.538 | 69.646 | 61.018 | 56.660 | 100.722 |
| Outpatient waiting time (median) | 33 | 36 | 50 | 36 | 34 | 42.5 |
| Pre-operative Oxford hip score |  |  |  |  |  |  |
| 0-4 | 0.017 | 0.017 | 0.019 | 0.019 | 0.016 | 0.023 |
| 5-8 | 0.081 | 0.080 | 0.085 | 0.088 | 0.084 | 0.103 |
| 9-12 | 0.136 | 0.140 | 0.147 | 0.140 | 0.144 | 0.171 |
| 13-16 (reference group) | 0.174 | 0.174 | 0.187 | 0.196 | 0.189 | 0.190 |
| 17-20 | 0.183 | 0.188 | 0.191 | 0.180 | 0.181 | 0.175 |
| 21-24 | 0.154 | 0.161 | 0.152 | 0.158 | 0.148 | 0.131 |
| 25-28 | 0.121 | 0.117 | 0.111 | 0.109 | 0.118 | 0.101 |
| 29-32 | 0.078 | 0.072 | 0.061 | 0.065 | 0.071 | 0.063 |
| 33-48 | 0.055 | 0.052 | 0.047 | 0.045 | 0.050 | 0.042 |
| Symptoms duration |  |  |  |  |  |  |
| Less than a year | 0.154 | 0.152 | 0.136 | 0.138 | 0.133 | 0.061 |
| 1-5 years (base) | 0.680 | 0.672 | 0.695 | 0.690 | 0.693 | 0.764 |
| 6-10 years | 0.109 | 0.117 | 0.112 | 0.113 | 0.115 | 0.119 |
| >10 years | 0.057 | 0.059 | 0.058 | 0.058 | 0.059 | 0.056 |
| Male (reference group: female) | 0.395 | 0.394 | 0.403 | 0.400 | 0.400 | 0.417 |
| Age |  |  |  |  |  |  |
| 18-44 | 0.015 | 0.013 | 0.016 | 0.017 | 0.015 | 0.019 |
| 45-54 | 0.076 | 0.077 | 0.085 | 0.090 | 0.084 | 0.098 |
| 55-64 | 0.220 | 0.220 | 0.231 | 0.224 | 0.238 | 0.258 |
| 65-74 (reference group) | 0.405 | 0.418 | 0.404 | 0.398 | 0.398 | 0.383 |
| 75-84 | 0.252 | 0.244 | 0.236 | 0.242 | 0.236 | 0.220 |
| >=85 | 0.032 | 0.028 | 0.029 | 0.029 | 0.029 | 0.023 |
| Ethnicity |  |  |  |  |  |  |
| White (reference group) | 0.789 | 0.757 | 0.743 | 0.740 | 0.702 | 0.556 |
| Mixed | 0.001 | 0.002 | 0.002 | 0.001 | 0.001 | 0.002 |
| Asian | 0.002 | 0.002 | 0.001 | 0.003 | 0.003 | 0.002 |
| Black | 0.002 | 0.001 | 0.002 | 0.002 | 0.003 | 0.002 |
| Other ethnicity | 0.001 | 0.001 | 0.001 | 0.001 | 0.001 | 0.002 |
| Missing Ethnicity | 0.205 | 0.237 | 0.251 | 0.253 | 0.290 | 0.436 |
| Rural (reference group: urban) | 0.335 | 0.341 | 0.348 | 0.314 | 0.306 | 0.313 |
| Emergency admissions^ |  |  |  |  |  |  |
| 0 (reference group) | 0.951 | 0.955 | 0.950 | 0.952 | 0.950 | 0.947 |
| 1 | 0.043 | 0.040 | 0.042 | 0.042 | 0.043 | 0.043 |
| >=2 | 0.006 | 0.005 | 0.008 | 0.007 | 0.007 | 0.010 |
| Primary diagnosis^^ |  |  |  |  |  |  |
| Coxarthrosis, unspecified (ref) | 0.879 | 0.871 | 0.874 | 0.873 | 0.852 | 0.893 |
| Other primary coxarthrosis | 0.053 | 0.052 | 0.051 | 0.032 | 0.032 | 0.011 |
| Polyarthrosis, unspecified | 0.026 | 0.030 | 0.035 | 0.059 | 0.079 | 0.066 |
| Osteonecrosis, unspecified | 0.003 | 0.003 | 0.003 | 0.003 | 0.004 | 0.004 |
| Primary coxarthrosis, bilateral | 0.002 | 0.003 | 0.005 | 0.005 | 0.005 | 0.001 |
| Secondary diagnosis^^^ |  |  |  |  |  |  |
| Hypertension, uncomplicated | 0.425 | 0.423 | 0.424 | 0.425 | 0.417 | 0.402 |
| Obesity | 0.157 | 0.197 | 0.266 | 0.246 | 0.256 | 0.246 |
| Chronic pulmonary disease | 0.118 | 0.120 | 0.129 | 0.131 | 0.136 | 0.112 |
| Diabetes, uncomplicated | 0.073 | 0.076 | 0.078 | 0.075 | 0.074 | 0.071 |
| Cardiac arrhythmias | 0.051 | 0.054 | 0.061 | 0.063 | 0.062 | 0.055 |
| Hypothyroidism | 0.064 | 0.069 | 0.082 | 0.085 | 0.086 | 0.080 |
| Depression | 0.049 | 0.054 | 0.066 | 0.075 | 0.088 | 0.085 |
| Renal failure | 0.043 | 0.045 | 0.077 | 0.076 | 0.066 | 0.062 |
| Rheumatoid arthritis | 0.030 | 0.033 | 0.043 | 0.042 | 0.043 | 0.041 |
| Valvular disease | 0.010 | 0.012 | 0.013 | 0.011 | 0.014 | 0.011 |
| IMD quintile |  |  |  |  |  |  |
| 1 (base – least deprived) | 0.256 | 0.255 | 0.259 | 0.267 | 0.266 | 0.269 |
| 2 | 0.255 | 0.261 | 0.261 | 0.263 | 0.263 | 0.267 |
| 3 | 0.218 | 0.225 | 0.215 | 0.209 | 0.208 | 0.211 |
| 4 | 0.167 | 0.162 | 0.165 | 0.153 | 0.159 | 0.145 |
| 5 (most deprived) | 0.104 | 0.097 | 0.101 | 0.108 | 0.103 | 0.109 |
| Referral source |  |  |  |  |  |  |
| GP (base) | 0.971 | 0.958 | 0.953 | 0.964 | 0.959 | 0.904 |
| Responsible consultant | 0.000 | 0.015 | 0.039 | 0.029 | 0.024 | 0.086 |
| Other | 0.029 | 0.027 | 0.008 | 0.007 | 0.017 | 0.009 |
| Number of appointments in spell |  |  |  |  |  |  |
| 1 (base) | 0.544 | 0.513 | 0.439 | 0.528 | 0.576 | 0.471 |
| 2 | 0.175 | 0.172 | 0.214 | 0.190 | 0.173 | 0.173 |
| 3 | 0.148 | 0.170 | 0.182 | 0.142 | 0.120 | 0.135 |
| >3 | 0.132 | 0.145 | 0.164 | 0.140 | 0.131 | 0.222 |

Notes.

^ Emergency admissions in the year preceding a hip replacement surgery.

^^ Indicators for the most frequent primary diagnosis at admission and an indicator for any other primary diagnosis. We only show the most common five primary diagnoses here, others included in the models are: other secondary coxarthrosis, other dyspalstic coxarthrosis, gonarthrosis (unspecified), arthritis (unspecified), other post-traumatic coxarth., other.

^^^ Indicators for each of the Elixhauser morbidities recorded in the year preceding a hip replacement surgery. We only show the most common ten secondary diagnoses here, others included are: fluid & electrolyte dis, alcohol abuse, other neurological dis., cong. heart failure, peripheral vascular dis., deficiency anemia, solid tumor w/o met., liver disease, coagulopathy, diabetes complicated, peptic ulcer, weight loss, pulmonary circulation disorder, metastatic cancer, lymphoma, drug abuse, paralysis, psychoses, hypertension (complicated), blood loss anaemia.

**Appendix E. Simulation**

Define $h_{i}$ as the health gain of patients in a given severity group *i*. This can be measured as the difference between post-operative health and pre-operative health on the EQ-5D scale. Define $w_{i}$ as the waiting time for this severity group. The total health benefit, measured as discounted QALYs, from a hip replacement for a patient in this group is:

$H_{i}{(w}_{i})= h_{i}\left( 1-w_{i} \right)+{\delta h}_{i}+\delta^{2}h_{i}+\ldots+\delta^{T-1}h_{i}=h_{i}\sum_{t=0}^{T-1} \delta^{t}-h_{i}w_{i}$,

where *T* is the length of the functioning of the hip replacement (measured in years, e.g. 10 years), and $\delta$ is the discount rate. We measure waiting time $w_{i}$ in years, and assume that it is always less than one year, i.e. $\in[0,1]$.

The total health gain for patients in this group is equal to:

$T_{i}{(w}_{i})= N_{i}H_{i}{(w}_{i})$,

where $N_{i}$ is the number of patients in this group. The total health gain across all patients is equal to:

$$T(w)=\sum_{i=1}^{I} T_{i}{(w}_{i}).$$

Suppose that the vector of waiting times$w_{i}$ across groups of patients with different pre-operative health changes, for example by reducing the waiting time for the group with lowest pre-operative health and increasing the waiting time of the group with highest pre-operative health. Then the effect on overall health is equal to:

$\Delta T=T(w^{A})-T(w^{B})=\sum_{i=1}^{I} N_{i}h_{i}(w_{i}^{A}-w_{i}^{B})$,

where $w_{i}^{A}$ and $w_{i}^{B}$ are the waiting time in the pre-policy period A, and in the post-policy period B. Given that the waiting time is less than one year, the overall health effect of re-prioritisation only depends on the health gains in the first year.

The above model relies on the assumptions that the number of patients is fixed and that waiting times do not affect the health gain from treatment. We can relax these assumptions by assuming that the health gain reduces with waiting time, $h_{i}{(w}_{i})$ with ${h'}_{i}{(w}_{i})<0$, and that demand for surgery reduces with waiting times, $N_{i}{(w}_{i})$ with ${N'}_{i}{(w}_{i})<0$. The health benefit from a hip replacement for a patient in severity group *i* is now given by:

$H_{i}{(w}_{i})=h_{i}{(w}_{i})[\sum_{t=0}^{T-1} \delta^{t}-w_{i}]$

and the total health gain across groups is given by

$T(w)=\sum_{i=1}^{I} N_{i}{(w}_{i})h_{i}{(w}_{i}) [\sum_{t=0}^{T-1} \delta^{t}-w_{i}]$.

If we compare the total health gain when waiting times are equal to $w_{i}^{A}$ in the pre-policy period and equal to $w_{i}^{B}$ in the post-policy period, the effect of this policy on total health gain is:

$$\Delta T=T(w^{A})-T(w^{B})$$

or, more explicitly,

$\Delta T=\sum_{i=1}^{I} N_{i}(w_{i}^{A})h_{i}(w_{i}^{A}) [\sum_{t=0}^{T-1} \delta^{t}-w_{i}^{A}]-\sum_{i=1}^{I} N_{i}(w_{i}^{B})h_{i}(w_{i}^{B}) [\sum_{t=0}^{T-1} \delta^{t}-w_{i}^{B}]$.

**Table A14. Simulation of health gain from additional prioritisation**

| OHS category | Number of patients | Prioritised Waiting Time  (-40%, +40%) | Change in waiting time from prioritisation | HRQoL gain per patient | Health (QALY) gain from prioritisation |
| --- | --- | --- | --- | --- | --- |
| 0-4 | 901 | 66 | -44 | 0.69 | 74.94 |
| 5-8 | 2,592 | 67 | -45 | 0.68 | 217.30 |
| 9-12 | 3,529 | 71 | -47 | 0.65 | 295.37 |
| 13-16 | 3,855 | 121 | 0 | 0.53 | 0 |
| 17-20 | 3,515 | 123 | 0 | 0.39 | 0 |
| 21-24 | 42 | 124 | 0 | 0.30 | 0 |
| 21-24 | 2,453 | 174 | 50 | 0.30 | -100.81 |
| 25-28 | 1,864 | 175 | 50 | 0.26 | -66.39 |
| 29-32 | 1,134 | 178 | 51 | 0.21 | -33.27 |
| 33-48 | 932 | 181 | 52 | 0.16 | -21.24 |
|  |  |  |  | **TOTAL** | **365.90** |

**Table A15. Simulation of health gain from additional prioritisation when waiting times reduce health**

| OHS category | Number of patients | Prioritised Waiting Time  (-20%, +20%) | Change in waiting time from prioritisation | HRQoL gain per patient | Health (QALY) gain from prioritisation |
| --- | --- | --- | --- | --- | --- |
| 0-4 | 901 | 88 | -22 | 0.689 | 37.41 |
| 5-8 | 2,592 | 90 | -22 | 0.679 | 106.05 |
| 9-12 | 3,529 | 94 | -24 | 0.649 | 150.53 |
| 13-16 | 3,855 | 121 | 0 | 0.530 | 0.00 |
| 17-20 | 3,515 | 123 | 0 | 0.390 | 0.00 |
| 21-24 | 334 | 124 | 0 | 0.300 | 0.00 |
| 21-24 | 2,495 | 149 | 25 | 0.299 | -51.04 |
| 25-28 | 1,864 | 150 | 25 | 0.259 | -33.02 |
| 29-32 | 1,134 | 152 | 25 | 0.209 | -16.21 |
| 33-48 | 932 | 155 | 26 | 0.159 | -10.53 |
|  |  |  |  | **TOTAL** | **183.19** |
